# Sexual dimorphism in the colonic microbiome and host’s transcriptomics profiles of a murine model of multiple sclerosis

**DOI:** 10.1016/j.clicom.2026.03.003

**Published:** 2026-04-06

**Authors:** William J. Doyle, Sean M. Schumacher, Megan R. Gates, Natalie Sofaly, Ella Angelo, Hannah Hedelius, David R. Johnson, Joshua Wells, Michael Perlmutter, Kacey Caradonna, Javier Ochoa-Repáraz

**Affiliations:** a Biomolecular Sciences Graduate Program, Boise State University, Boise, ID 83725, USA; b Department of Biological Sciences, Boise State University, Boise, ID 83725, USA; c Computing Ph.D. Program, Boise State University, Boise, ID 83725, USA; d Biology, Brigham Young University–Idaho, Rexburg, ID 83460, USA; e Department of Mathematics, Boise State University, Boise, ID 83725, USA; f Hooke Laboratories LLC, 439 S Union St, Lawrence, MA 01843, USA

**Keywords:** Microbiome, Transcriptomics, Colon, Inflammation, EAE, MS

## Abstract

**Background:**

Multiple Sclerosis is a chronic autoimmune disease that attacks the myelin sheath in the central nervous system, with a higher prevalence among female patients. We and others have documented significant changes in microbial taxa in response to the induction of active experimental autoimmune encephalomyelitis (EAE), an MS model.

**Objective:**

To evaluate sex as a biological variable in both the host and colonic microenvironment during active EAE.

**Methods:**

We conducted colonic transcriptomics and microbiota analysis of colonic fecal content in male and female EAE C57BL/6 J mice and controls at the time of disease induction, pre-onset, and peak disease.

**Results:**

Analysis showed significant sex-specific differences in colonic gene expression during EAE. As disease severity increased, the profiles of colon microbiome and transcriptomics became less distinct.

**Conclusions:**

Our results suggest early changes in colonic inflammatory pathways, with notable differences between males and females associated with microbiota alterations triggered by disease induction.

## 1. Introduction

Multiple Sclerosis (MS) is a chronic autoimmune disorder that causes degradation of the myelin sheath in the central nervous system (CNS). Disease incidence of MS relevance has increased by 30 % since 2013, with currently 1 million cases being in the US and ~3 million people globally presently diagnosed [1–5], with a prevalence of >300 individuals per 100,00 US residents reported [5,6]. While the etiology of MS is not fully understood, genetic and environmental factors are believed to play a role. The hallmark symptoms of MS include dizziness, fatigue, and gait problems, progressing to visual impairment, muscle loss, paralysis, and incontinence [7–9]. As it has been documented for other autoimmune diseases, there are 2–3 female MS patients for every male, a ratio that has also been reported to be on the rise over the last few decades [6].

Growing evidence supports that the combined genetic pool of intestinal microbes (termed the gut microbiome) is a key influence on disease incidence and symptom progression. The gut microbiome composition is significantly different in MS patients (relapse and remission) and healthy individuals, in both males and females [3, 10–13]. In experimental autoimmune encephalomyelitis (EAE), the most used animal model to study MS, disease induction modifies the gut microbiome [14–17]. Due to non-invasiveness, most clinical and experimental studies rely on secreted fecal samples to represent the gut microbiome composition. However, it has been demonstrated that fecal and mucosal-associated microbiota differ significantly [18–22]. Additionally, we and others have shown that the gut microbiome modifications and microbial products can be immunomodulatory in EAE and comparable animal models, while changes in the microbiome composition are associated with increased intestinal permeability and immune cell infiltration in the CNS [15,23–25]. Evidence supports that reduced intestinal barrier integrity characterizes EAE/MS pathology and severity, and other autoimmune diseases [26–28]. Not considering spontaneous models of the disease, EAE can be induced in mice actively (active EAE) by the administration of a self-peptide emulsified in complete Freund’s adjuvant (CFA) and two doses of pertussis toxin (PTX) or passively (passive EAE) by adoptive transfer of encephalitogenic CD4^+^ T cells [29]. Despite the need for CFA and PTX, the literature on EAE predominantly comprises studies conducted by active immunization, including those assessing the microbiome. Thus, given the model’s widespread use in academic and drug-efficacy experiments, it is relevant to assess the effects of active EAE induction and the required reagents on the gut microenvironment.

Here, we present a descriptive study analyzing the proximal colonic transcriptomic (RNA-seq) and colonic microbiome (V3-V4 16S rRNA amplicon sequencing) profiles of male and female mice with EAE across the disease course. To quantify colon-microbiome-to-transcriptomic changes in the colon, we used an integrative mathematical analysis of raw colonic RNA-seq data and 16S rRNA sequences. Our findings show significant differences in the colon transcriptomics profile and colon microbiome throughout EAE at each time point and between males and females. Furthermore, the results show that remarkable changes in the host’s genetic profile and colon microbiome occur before the first clinical scores even appear in mice.

## 2. Materials and methods

### 2.1. Animal housing conditions

Ten-week-old male and female C57BL/6 J mice were purchased from Jackson Labs (Bar Harbor, ME), with females weighing ~20 g and males weighing ~25 g. Animals were housed in groups of five in individually vented cages on a 12-hour light and dark cycle (22 ± 1 °C; 45 % ± 10 % humidity). Animals had access to food and water ad libitum, in accordance with Boise State University policies and protocols for animal well-being and overall health (IACUC #AC23–034).

### 2.2. EAE induction and clinical evaluation

Animals were left in their shipping containers with food and hydrogel for 3 days. Next, they were placed in appropriate groups (*n* = 3–5 per cage, all-male/all-female, respectively) and given one week to acclimate to BSU housing conditions. All experimental groups were housed in the same room of the animal facility under identical controlled conditions throughout the EAE experiments. EAE was induced using Hooke Laboratories induction kits (Hooke Kit^™^ EK-2110, Hooke Laboratories, Lawrence, MA). MOG_35–55_ emulsified with complete Freund’s adjuvant (CFA) in pre-loaded syringes/needles with Pertussis toxin (PTX) in a glycerol buffer in separate tubes. Animals were anesthetized with 2.0 % isoflurane for ~5 min, then given 0.2 mL MOG_35–55_ emulsified in CFA subcutaneously on day 0 (0.1 mL along the midline of the upper and lower back). Approximately 6 h after MOG_35–55_ injection on day 0, mice received 110 ng of PTX intraperitoneally (diluted in phosphate-buffered saline), and the same PTX dose was administered 24 h later. Sham groups were given CFA and PTX doses described above. Groups of 7–9 female and male mice were used in this study. Two independent EAE studies were performed. The number of mice in the first experiment, where the colon tissue, adjacent colon fecal sample, and histology were analyzed by RNA-seq and 16S, are as follows: Female CFA+PTX (*n* = 7); female naïve (pre-induction of disease; D0, *n* = 7); female pre-onset EAE (*n* = 7); female peak EAE (*n* = 9); male CFA+PTX (*n* = 7); male naïve (pre-induction of disease; D0, *n* = 7); male pre-onset EAE (*n* = 7); male peak EAE (*n* = 9). In the second EAE experiment (samples not part of analysis but used as experimental repeat for EAE severity and progression), animals were organized as follows: male EAE (*n* = 10); female EAE (*n* = 10). Combined, the number of mice used in the EAE experiments was the following: Female EAE (*n* = 14); Female CFA+PTX (*n* = 7); Male EAE (*n* = 19); Male CFA+PTX (*n* = 7).

Mice were scored according to Hooke Laboratories on a 0–5 scale starting on day 7 and daily until the group was sacrificed: 0- no detectable signs of EAE, 0.5- distal end of the tail is limp, 1.0- tail is entirely limp, 1.5- hind limb weakness with completely limp tail, 2.0- partial unilateral hind limb paralysis, 2.5- partial bilateral hind limb paralysis, 3.0- bilateral complete hind limb paralysis, 3.5- bilateral complete hind limb paralysis accompanied with partial front limb paralysis, 4.0- quadriplegia. When mice exhibited a clinical score of 2.5 (unable to eat/drink from a raised food cage and water bottle), a shallow cup was placed with water-soaked food in the bedding. Body weights were collected weekly and compared with initial weights (100 %), expressed as a percentage of the initial body weight at that time point. Animals were euthanized at timepoints 0 (baseline measurements at pre-induction at day 0 (D0)), 9 (pre-onset of disease; D9), and at the peak of disease groups (days 17 and 19 for females and males, respectively, when the group average reached a clinical score of 2.5 to mitigate animal distress and not lose animals throughout the study).

### 2.3. Colonic tissue and fecal collection

Euthanized mice were sprayed thoroughly with 70 % ethanol and laid on their back with their arms and legs pinned to splay outward. An incision was made into the skin from the anus to the throat without disrupting the peritoneum. The skin was held away from the abdomen with pins, and the peritoneum was sprayed thoroughly with 70 % ethanol. Fresh, sterile scissors were used to make an incision up the center of the peritoneum from just above the anus to the sternum. Three centimeters of the distal end of the colon were removed and placed on a clean paper towel soaked in sterile PBS (pH ~7.4). The colon was cut open lengthwise. The entire length of the extracted colon and fecal samples was collected from the distal end of the colon. The remaining feces were removed, and the colons were cut into three 1 cm sections. Each section was flushed clean with sterile PBS. Colons and fecal samples within colons from all experimental groups of mice were collected at the same time across the described experimental timepoints.

### 2.4. Colonic histological analysis

One cm distal sections of the colons were placed into microcassettes and then submerged into ~20:1 vol (fixative: tissue) of 10 % neutral buffered formalin (NBF) solution. Tissues were fixed in solution for 12–14 h. Fixed tissues were removed from the NBF solution, washed with 100 % ethanol, and then with 70 % ethanol. Washed tissues were then submerged in ~20:1 vol of 70 % ethanol and stored in a parafilm-sealed container at 4 °C until processed. Hooke Laboratories performed histology sample preparation, staining, and analysis.

### 2.5. RNA-seq Transcriptomics Analysis

#### 2.5.1. Tissue preparation

Colon tissue used for RNA sequencing was collected from the section of the colon 2 cm distal to the anus. After cleaning as described above, tissues were placed into cryopreservation tubes and submerged in liquid nitrogen for snap freezing. Tubes were removed from liquid nitrogen and stored at −80 °C until ready for processing.

#### 2.5.2. RNA extraction and sequencing

Colon tissue samples were sent to LC Sciences, LLC (Houston, TX, USA) for RNA extraction, sequencing, and bioinformatics analysis. Poly (A) RNA sequencing library was prepared following Illumina’s TruSeq stranded-mRNA sample preparation protocol. RNA integrity was assessed using the Agilent Technologies 2100 Bioanalyzer. Poly(A) tails containing mRNAs were purified using oligo-(dT) magnetic beads with two rounds of purification. Purified poly(A) RNA was fragmented using a divalent cation buffer at elevated temperature. Quality control analysis and quantification of the sequencing library were performed using Agilent Technologies 2100 Bioanalyzer High Sensitivity DNA Chip. Paired-end sequencing was performed on Illumina’s NovaSeq 6000 sequencing system.

#### 2.5.3. Transcripts assembly

Cutadapt [30] and Perl scripts (LC Sciences, LLC, Houston, TX, USA) were used to remove low-quality base calls, undetermined base calls, and reads with adaptor contamination. Sequence quality was verified using FastQC (https://www.bioinformatics.babraham.ac.uk/projects/fastqc/). HISAT2 [31] was used to map reads to a reference genome (ftp://ftp.ensembl.org/pub/release101/fasta/mus_musculus/dna/), and these mapped reads were assembled using StringTie [32]. All transcriptomes were merged to reconstruct a comprehensive transcriptome using Perl scripts and Gffcompare. After the final transcriptome was generated, Stringtie [32] and ballgown (https://www.bioconductor.org/packages/release/bioc/html/ballgown.html) were used to estimate expression levels of all transcripts.

#### 2.5.4. Differential expression analysis

Differential expression analysis of mRNA between two groups was performed using R and the edgeR [33] and DESeq2 package [34]. The mRNAs with a false discovery rate (FDR) below 0.05 and an absolute fold change of 2 or greater were considered differentially expressed genes. Top ten differentially expressed genes ordered by FDR significance compiled into a table using the knitr R package (v1.50) [35].

#### 2.5.5. Functional analysis

Kyoto Encyclopedia of Genes and Genomes (KEGG) pathway and Gene Ontology (GO) enrichment visualized using gene-level outputs provided by LC Sciences. Summarization and visualization were performed using the clusterProfiler (v4.16.0) [36] and enrichplot (v1.28.4) [37] packages in R. Background sizes were derived from the Bioconductor package org.Mm.eg.db annotation database and KEGG database for *Mus musculus*. Significance was determined using the Benjamini-Hochberg (BH) method for FDR bias correction at *q* < *0.05* with extremely low p-values floored at 1 * 10^−300^.

### 2.6. Gut microbiome analysis

#### 2.6.1. DNA extraction and 16 s rDNA sequencing

Fecal samples were sent to LC Sciences, LLC (Houston, TX, USA) for 16 s rDNA extraction, sequencing, and bioinformatics analysis. DNA was extracted from fecal samples, and the 341F/805R primers were used to amplify the V3 and V4 regions of 16S rDNA, producing an amplicon of approximately 465 bp. The amplified library was sequenced on a NovaSeq platform with 250 bp paired-end reads.

#### 2.6.2. 16 s rDNA sequence cleaning and filtering

Raw FASTQ files underwent read merging by overlapping sequences, data quality control, and chimera filtering, resulting in high-quality clean data. Divisive Amplicon Denoising Algorithm (DADA2) [38] was used for dereplication and generation of representative sequences at single-base resolution yielding Amplicon Sequence Variant (ASV) tables. The resulting characteristic representative ASV sequences were used for downstream analyses.

#### 2.6.3. Bioinformatic analysis

Alpha and beta diversity were calculated and visualized in R using the QIIME2 package after rarefying all samples to an equal sequencing depth. Then, according to SILVA (release 132) classifier, feature abundance was normalized using the relative abundance of each sample. Blast was used for sequence alignment [39], and feature sequences were annotated with the SILVA database for each representative sequence. Differential abundance of bacterial genera was performed using an Analysis of Composition of Microbiomes with Bias Correction 2 (ANCOM-BC2) using the ANCOMBC package (v2.10.1) [40] in R (v4.5.0). ASV count data were aggregated to the genus level using the phyloseq R package (v1.52.0) [41], and sex or experimental timepoint was modeled as a fixed effect. Analyses were run separately for each comparison, including between sex at each timepoint and within-sex comparisons across timepoints. Differentially abundant genera were visualized as forest plots using the ggplot2 R package (v4.0.0) [42] and significance was determined using the BH method [43] for bias correction at *q* < *0.05*. Firmicutes to Bacteroidota (F/B) ratios were calculated using phylum-level bacterial counts aggregated from ASV data using phyloseq. A pseudocount of 1 was applied to the numerator and denominator when calculating F:B ratio to avoid division by zero. Sex and timepoint-specific comparisons were assessed with two-sided Mann-Whitney U tests and visualized using GraphPad Prism (v10.6.1). Significance was determined at *p < 0.05; **, *p* < *0.01*; ***, *p* < 0.001.

### 2.7. RNA-seq-16S rRNA concatenated visualization

RNA or DNA sequence count features were transformed using the variance stabilizing transformation (VST) implemented in the R package DESeq2 [34]. We next min-max scaled and median-centered each feature (DNA or RNA sequence) onto the real interval [−0.5, 0.5] to give each feature equal distance weighting in the high-dimensional feature space for PHATE Algorithm analysis [44]. Finally, the PHATE algorithm was applied twice to each of the colon RNA sequence data alone, the fecal microbiome DNA sequence data alone, and the concatenated RNA and DNA sequencing data to obtain both a two-dimensional and three-dimensional reduction. Finally, we used the PHATE algorithm to produce two-dimensional and three-dimensional visualizations of the colon RNA sequences, the fecal microbiome DNA sequences, and the concatenated RNA and DNA sequencing data.

### 2.8. Statistical analysis for in vivo studies

For EAE clinical scores and percent body weight vs. initial (100 %) statistical analysis, Two-way ANOVA and Sidak’s multiple comparisons test were used. The Mann-Whitney test was used for statistical analysis of disease onset and severity index. *, *p* < *0.05*; **, *p* < *0.01*; ***, *p* < 0.001. Additionally, BioRender (biorender.com) was used to create a graphic abstract of the mouse GI tract. Sample size: EAE Female (*n* = 14); EAE Male (*n* = 19); CFA+PTX Female (*n* = 7); CFA+PTX Male (*n* = 7).

## 3. Results

### 3.1. EAE induction and progression in male and female mice

**Fig. 1.**
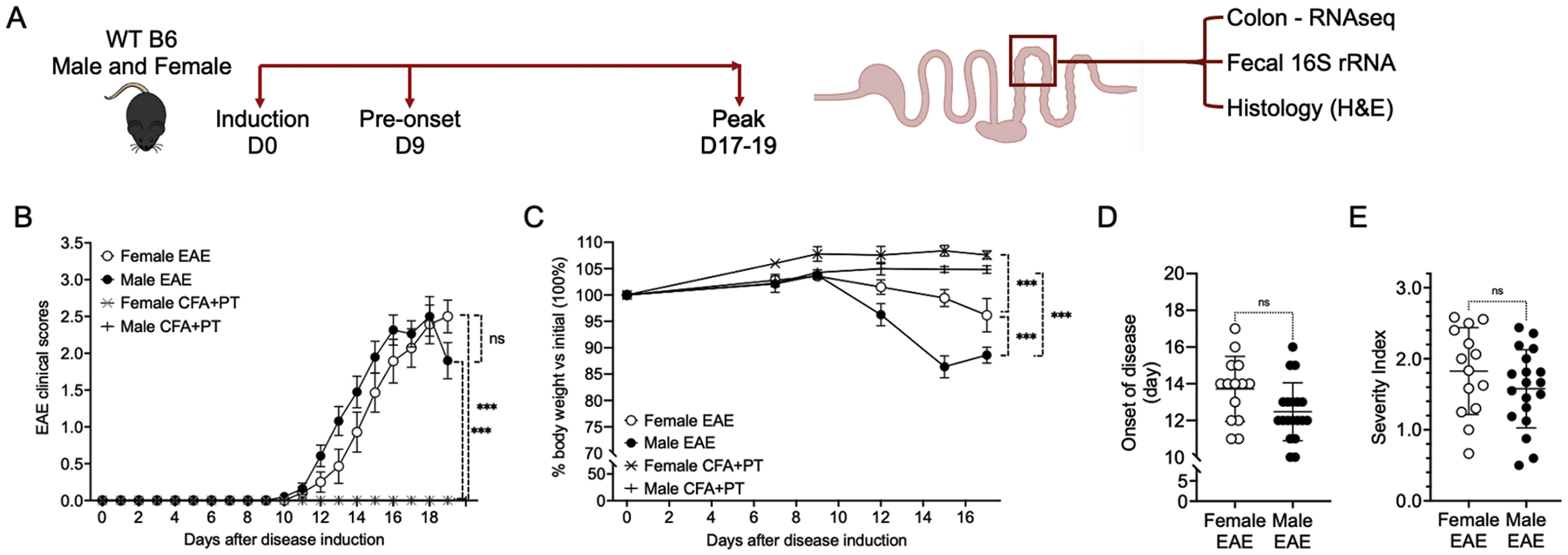
Evaluation of active EAE in male and female C57BL/6 (B6) wild-type (WT) mice. A) Experimental plan: EAE was actively induced on day 0. Colon samples and lumen fecal contents were collected on day 0, day 9 (before EAE symptoms appeared), and at the peak of the disease (day 19 for males and day 17 for females). Colon and lumen fecal samples from mice treated with CFA and PTX were collected on day 19. B) EAE clinical scores in female and male mice. C) EAE clinical scores in female and male mice. D) Disease onset. E) Severity index. F) Body weight of female mice relative to initial weight (100 %). G) Body weight of male mice relative to initial weight (100 %). B and C: Two-way ANOVA followed by Sidak’s multiple comparisons test. D and E: Mann-Whitney test. *, *p* < 0.05; **, *p* < 0.01; ***, *p* < 0.001. Mouse GI tract illustration created with BioRender. Sample sizes: Female EAE (*n* = 14); Female CFA+PTX (*n* = 7); Male EAE (*n* = 19); Male CFA+PTX (*n* = 7).

Ten-week-old female and male C57BL/6 mice were then used for an active EAE experiment (Fig. 1). The experimental approach and timeline for the study, including baseline D0 timepoint, are shown in Fig. 1A. Disease progression in males and females did not significantly differ (Fig. 1B). EAE induced significant reductions in body weights in both males and females compared to their respective control (*p* < 0.001 for both sexes), with EAE-males losing a higher percentage of body weight percentage compared to EAE-females (*p* < 0.001) (Fig. 1C). No significant differences in the onset of disease were found between male and female mice (Fig. 1D). There was no significant difference in disease severity, with female EAE reaching a severity index of 1.8 and 1.6 for male EAE (Fig. 1E). Additionally, there was no significant differences in colon histology analysis between sexes, or throughout disease progression (Supplementary Figure 1A–D). Overall, as expected for the C57BL/6 strain, no differences in the EAE profile of male and female mice were observed (Fig. 1B–E).

### 3.2. Male and female colon microbiome and transcriptomic profile at EAE induction

**Fig. 2.**
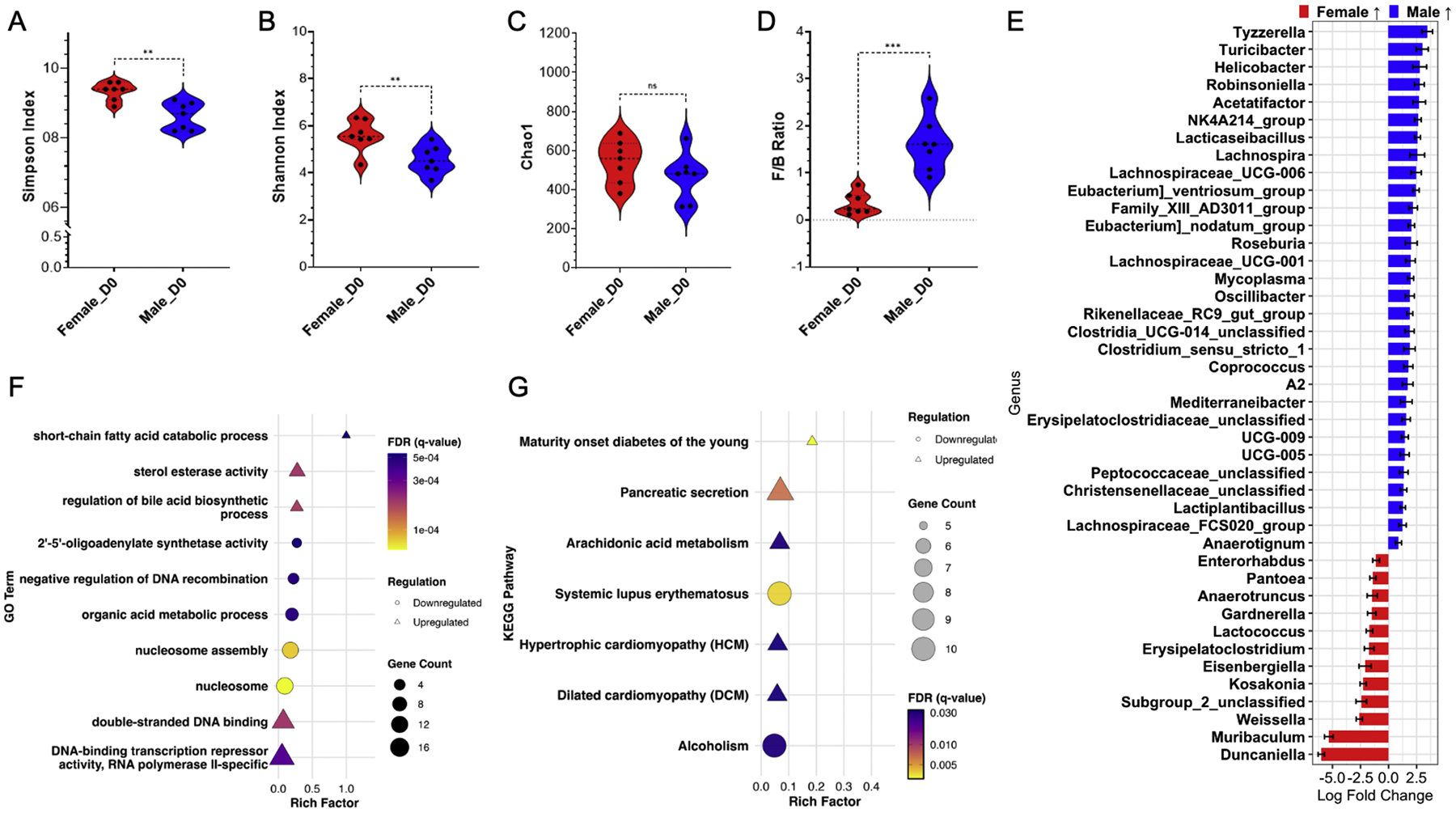
Comparison of male and female EAE mice colon microbiome and RNA-seq of adjacent colon section on day 0 (D0) of EAE induction. A) Simpson Index; B) Shannon Index; C) Chao1 Index; D) Firmicutes to Bacteroidetes ratio in colon fecal samples at D0; Mann-Whitney test: *, *p* < 0.05; **, *p* < 0.01; ***, *p* < 0.001. E) Differentially abundant genera in colon fecal samples on D0, comparing male and female mice. Positive log_2_ fold changes indicate genera enriched in males; negative values indicate enrichment in females. Only genera with a false discovery rate (FDR) < 0.05 are shown. Male vs. female D0 RNA-seq analysis using GO (F) and KEGG (G). Only significant enrichments with FDR < 0.05 are displayed. The shape of the point indicates regulation (triangle: upregulation; circle: downregulation), the size corresponds to gene count, and the color indicates FDR value. Sample size: female naïve (pre-induction; D0, *n* = 7); male naïve (pre-induction; D0, *n* = 7).

We first compared baseline differences in the colon microbiome of 10-week-old male and female C57BL/6 mice at EAE induction or sham treatment (CFA+PTX), used in animal autoimmune disease models [45, 46]. Animals were randomly divided into groups before a week of acclimation period. Next, mice were administered MOG_35–55_ and CFA+PTX, or CFA+PTX alone, with experimental and sham mice housed in the same room and under identical controlled housing conditions. Stool samples were collected from all animals, and groups were named according to the injections administered and labeled as D0 (pre-induction) for baseline comparisons. RNA-seq and 16S sequencing were performed on D0 and collected before EAE induction in all experimental groups at this timepoint. Males had a higher Firmicutes-to-Bacteroidetes (F/B) ratio at baseline than females (Fig. 2D), consistent with previous work analyzing secreted fecal samples in the EAE model [47,48]. Additionally, we found significant differences at baseline in the Simpson and Shannon Indices, but not in Chao1 (Fig. 2A–C and Supp. Fig. 2A). Alpha diversity indices (Simpson, Shannon, and Chao1) for all groups and time points are shown in Supplementary Figure 2. At the genus level, males had increased relative abundance of *Tyzzerella, Turicibacter, Helicobacter, Acetatifactor*, and NK4A214_group (*Ruminococcaceae*) (Fig. 2E). In addition, we found males had increased relative abundance of various *Lachnospiraceae* subtypes compared to baseline females; specifically, *Lachnospiraceae*_UCG-006, *Lachnospiraceae*_UCG-001, and *Lachnospiraceae*_FCS020_group (Fig. 2E). Females at D0, compared to pre-onset EAE also had higher levels of *Lachnospiraceae*_unclassified, though was not found in males at the same timepoint (Supplementary Fig. 3A/D). Interestingly, another group recently reported that *Lachnospiraceae* from the ileum of MS twins induce an EAE-like disease in transgenic TCR MOG_96–105_ mice in germ-free conditions, but no mice developed disease when treated with fecal material from the healthy twin [17]. Comparatively, females had a noticeable increase in relative abundance of the genus *Duncaniella* and *Muribaculum* in the colon compared to the colon of baseline males (Fig. 2E). Additionally, compared to baseline males, baseline females had increased relative abundance of the genus *Weissela, Kosakonia*, and *Eisenbergiella* (Fig. 2E). Of note, *Eisenbergiella* was also a dominant genus from the ileum of MS twins, and also relevant in inducing an EAE-like disease in the study previously mentioned [17].

We then investigated transcriptomic changes in the colon following EAE induction using RNA-seq and the Gene Ontology (GO) and Kyoto Encyclopedia of Genes and Genomes (KEGG) databases [49,50]. GO analysis highlighted that compared to females at baseline, males exhibited upregulation of pathways involving DNA binding, short-chain fatty acid catabolic process, sterol esterase activity, and regulation of bile acid biosynthetic process (Fig. 2F). KEGG analysis found at baseline, male colons had upregulation of pathways associated with maturity onset diabetes of the young, pancreatic secretion, arachidonic acid metabolism, and hypertrophic/dilated cardiomyopathy (Fig. 2G). Additionally, males at baseline had downregulation of pathways involving organic acid metabolic process and nucleosome (assembly) according to GO (Fig. 2F); KEGG highlighted baseline males had downregulation of pathways associated with systemic lupus erythematosus and alcoholism compared to baseline females (Fig. 2G). Interestingly, both males and females at D0 showed downregulation of extracellular matrix/region, cell adhesion molecules (CAMs), and *Staphylococcus aureus* infection compared to pre-onset EAE (Supp. Fig. 3 B/C and E/F). Overall, the colonic microenvironment differs between male and female mice, with intrinsic changes that occur upon EAE induction. Histologically, no significant differences were observed in colon sections of healthy male and female mice (Supp. Fig. 1).

### 3.3. Male and female colon microbiome and transcriptomics at pre-onset EAE

**Fig. 3.**
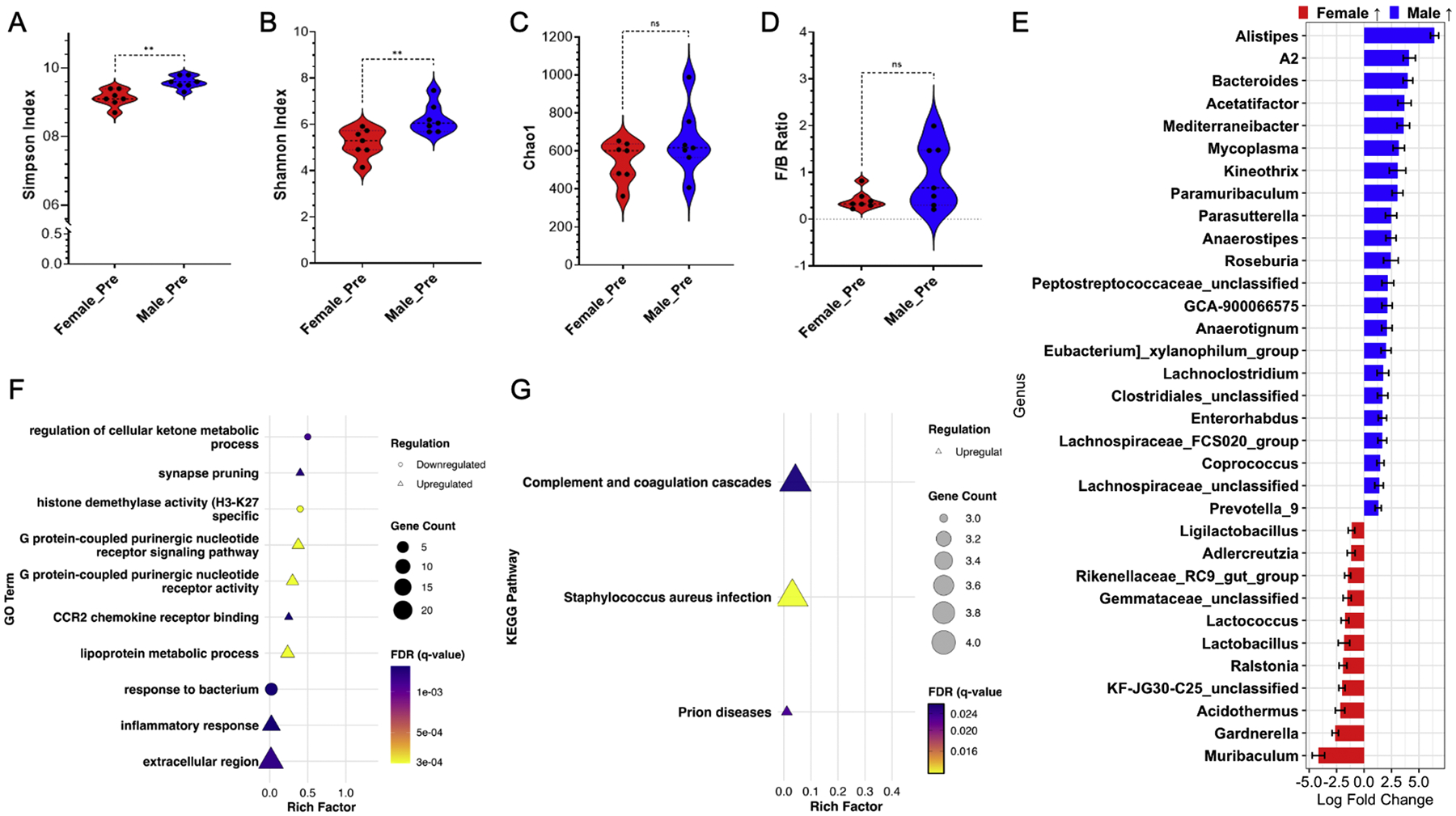
Comparison of male and female EAE mice colon microbiome and RNA-seq of adjacent colon section at pre-onset EAE (D9), post-induction of EAE. A) Simpson Index; B) Shannon Index; C) Chao1 Index; D) Firmicutes to Bacteroidetes ratio of colon fecal samples at D9 timepoint; Mann-Whitney test: *, *p* < 0.05; **, *p* < 0.01; ***, *p* < 0.001. E) Differentially abundant genera of colon fecal samples on D9, comparing male and female mice. Positive log_2_ fold changes indicate genera enriched in males, and negative values indicate enrichment in females. Only genera with a false discovery rate FDR < 0.05 are shown. Male vs. female at D9. RNA-seq analysis using GO (F) and KEGG (G). Only significant enrichment, FDR < 0.05, was displayed. The shape of the point indicates regulation (triangle: upregulation; circle: downregulation), the point size corresponds to gene count, and the point coloration corresponds to the FDR value. Sample size: Female pre-onset EAE (*n* = 7); Male pre-onset EAE (*n* = 7);.

Next, we investigated changes in the colon microbiome in male and female mice prior to the onset of EAE symptoms. Mice were euthanized on D9, since previous work from our lab found that the first signs of EAE symptoms occur on D10–11 [15,23]. At pre-onset EAE, the colon microbiomes of males and females showed no significant differences in F/B ratio, although males had greater variation than females (Fig. 3D), with significant differences in Simpson and Shannon Indices but not in Chao1 (Fig. 3A–C). Pre-onset EAE males had significant differences in Simpson and Shannon Indices compared to D0 and peak, although this was not observed in females (Supp. Fig. 2A–C). At the genus level, compared to pre-onset females, pre-onset males had increased relative abundance of *Alistipes*, A2, *Bacteroides*, Clostridiales_unclassified, and *Mediterraneibacter* (Fig. 3E). Pre-onset males also had increased relative abundance of *Anaerostipes* and *Roseburia* (Fig. 3E), which are all short-chain fatty acid (SCFA) producers, which have anti-inflammatory properties [51,52]. Additionally, pre-onset males had increased relative abundance of *Lachnospiraceae*_FCS020 and *Lachnospiraceae*_unclassified (Fig. 3E), following what was seen at baseline (Fig. 2E). Comparatively, pre-onset females had increased relative abundance of the genus *Muribaculum, Lactobacillus*, and *Lactococcus* (Fig. 3E).

At pre-onset EAE, we found significant differences in the colon transcriptomics between males and females before EAE symptoms occurred. GO analysis showed that pre-onset males had downregulation of pathways associated with the regulation of cellular ketone metabolic process and response to bacterium compared to pre-onset females (Fig. 3F). Downregulation of histone demethylase activity (H3-K27 specific) was also reported (Fig. 3F), but sexual dimorphism regarding this has already been described [53]. In addition, compared to pre-onset females, pre-onset males had upregulation of pathways corresponding to G protein-coupled purinergic nucleotide receptor signaling pathway and activity, lipoprotein metabolic process, and extracellular region (Fig. 3F). Interestingly, GO found males had upregulation of CCR2 chemokine receptor binding and inflammatory response (Fig. 3F); KEGG analysis found pre-onset males had upregulation of genes associated with complement and coagulation cascades, *Staphylococcus aureus* infection, and prion disease compared to pre-onset females (Fig. 3G). Differentially expressed genes between males and females at D0, pre-onset EAE, and peak EAE are shown in Table 1. Colonic microbiome differential abundance (genus) and colon RNA-sequencing, comparing D0 to pre-onset EAE in males and females, are shown in Supplementary Fig. 4. Overall, we found that both males and females had increased colonic inflammation and changes in the colon microbiome compared with their respective baselines. However, sexual dimorphism remained evident in colon microbiome composition and inflammatory pathways, indicating that the sexual dimorphism in the colon microenvironment undergoes key changes before the onset of EAE symptoms.

### 3.4. Male and female colon microbiome at peak EAE

**Fig. 4.**
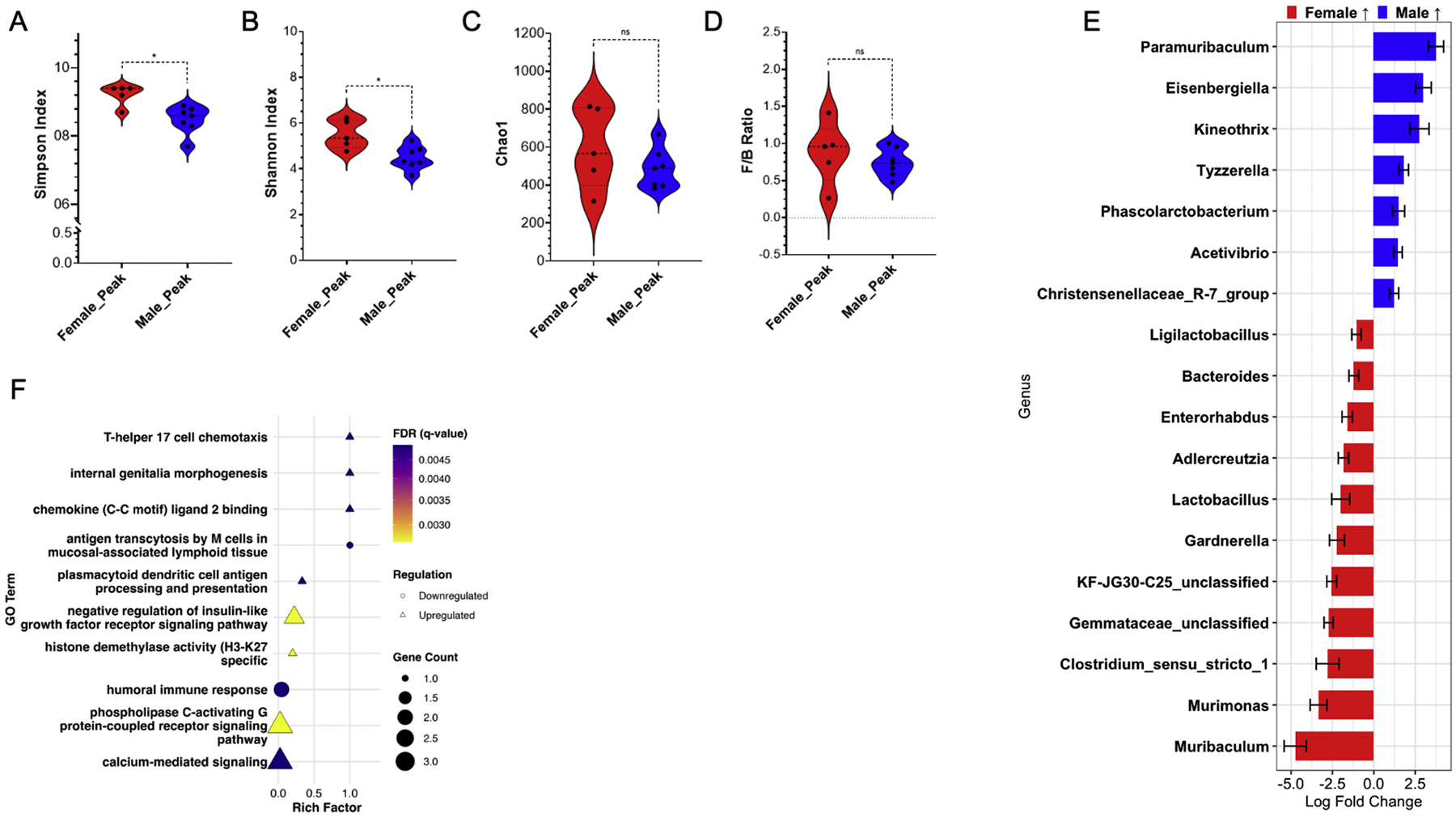
Comparison of the gut microbiome in male and female EAE mice and RNA-seq analysis of the adjacent colon section at peak EAE (D17/19 females/males). A) Simpson Index; B) Shannon Index; C) Chao1 Index; D) Firmicutes to Bacteroidetes ratio in colon fecal samples at peak EAE; Mann-Whitney test: *, *p* < 0.05; **, *p* < 0.01; ***, *p* < 0.001. E) Differentially abundant genera in colon fecal samples at peak EAE, comparing males and females. Positive log_2_ fold changes show genera enriched in males, negative values indicate enrichment in females. Only genera with a false discovery rate (FDR) < 0.05 are displayed. F) RNA-seq analysis comparing male and female mice at peak EAE using GO; only significant enrichments (FDR < 0.05) are shown. The shape of each point indicates regulation (triangle: upregulation; circle: downregulation), point size reflects gene count, and color indicates FDR value. No significant KEGG pathways are observed between the female and male peak EAE groups. Sample sizes: Female peak EAE (*n* = 9); Male peak EAE (*n* = 9).

We then investigated the transcriptomic and colon microbiome composition at peak EAE. No significant differences in F/B ratio or Chao1 in the colon of male and female mice at peak EAE (Fig. 4C/D), though there were significant differences in Simpson and Shannon indices (Fig. 4A/B). At the genus level, peak EAE males had increased relative abundance of the genus *Paramuribaculum, Eisenberiella, Kineothrix, Tyzzerella, Phascolarctobacterium, Acetivibrio*, and Christenselellaceae_R-7_group (Fig. 4E). Females at peak EAE had increased relative abundance of the genus *Muribaculum, Murimonas*, Clostridium_sensu_stricto_1, *Lactobacillus, Bacteroides*, and *Ligilactobacillus* compared to males at peak EAE (Fig. 4E).

RNA-seq analysis of the colon at peak EAE revealed less pronounced differences compared to baseline and pre-onset EAE between male and female mice. Notably, KEGG found no significant difference in peak EAE between males and females. GO highlighted peak EAE males had upregulation of genes associated with T-helper 17 cell chemotaxis, chemokine (C–C motif) ligand 2 binding, and plasmacytoid dendritic cell antigen processing and presentation (Fig. 4F). In addition, peak EAE males had downregulation of antigen transcytosis by M cells in mucosal-associated lymphoid tissue and humoral immune response (Fig. 4F). Interestingly, males had upregulation of pathways associated with negative regulation of insulin-like growth factor receptor signaling pathway, phospholipase C-activating G protein-coupled receptor signaling pathway, and calcium mediated signaling compared to peak EAE females (Fig. 4F). Differentially expressed genes between males and females at D0, pre-onset EAE, and peak EAE can be seen in Table 1. Overall, the differences in the colon microbiome and transcriptomics between males and females at the peak of EAE are less apparent than at baseline, indicating that EAE mitigates the sexual dimorphism in the colon microbiome.

### 3.5. Comparison between EAE and CFA+PTX treatments in male mice

**Fig. 5.**
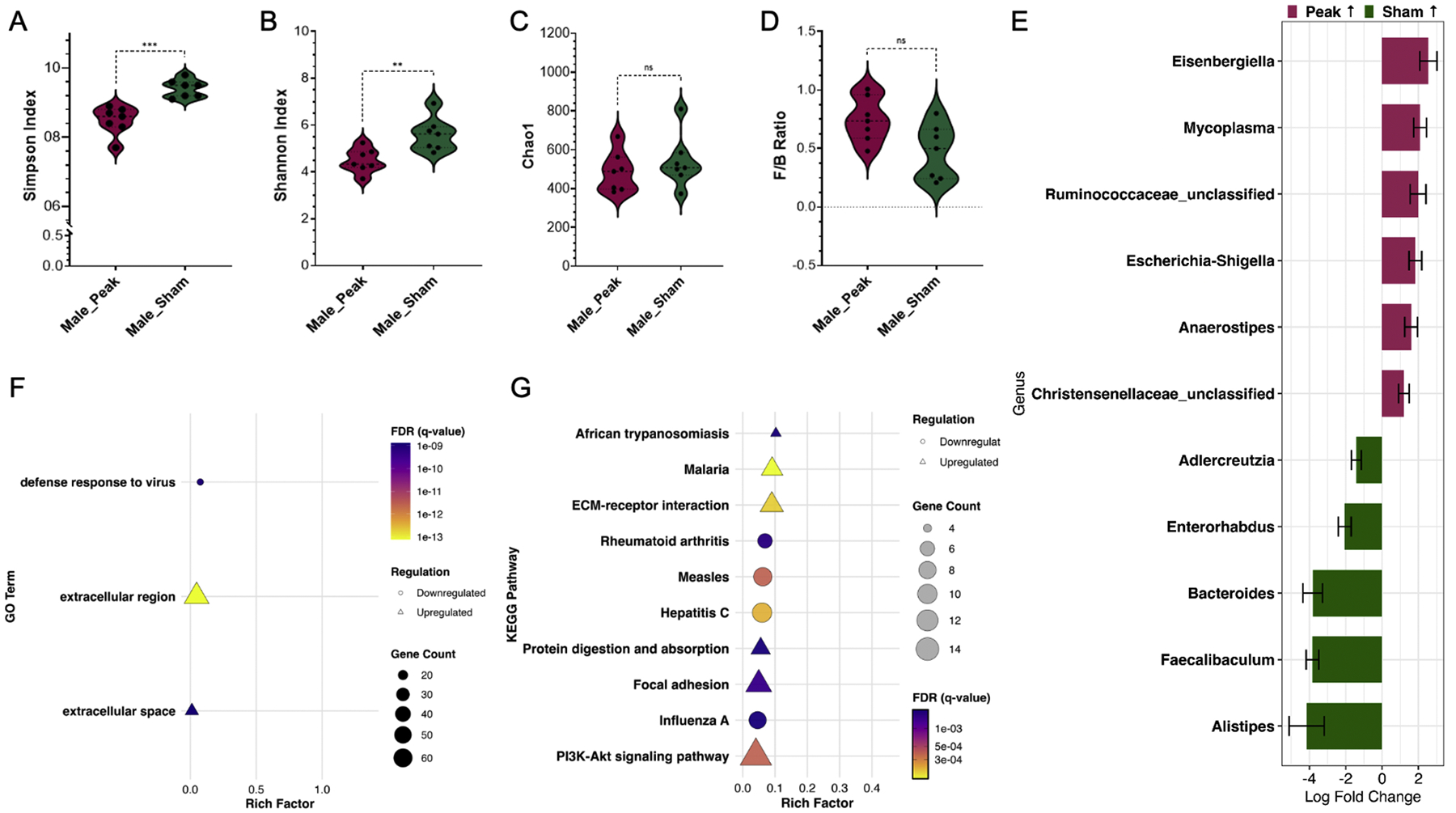
Comparison of male colon microbiome and RNA-seq of adjacent colon section at peak EAE versus male CFA+PTX. A) Simpson Index; B) Shannon Index; C) Chao1 Index; D) Firmicutes to Bacteroidetes ratio in colon fecal samples comparing male peak EAE to male CFA+PTX group; Mann-Whitney test: *, *p* < 0.05; **, *p* < 0.01; ***, *p* < 0.001. E) Differentially abundant genera in colon fecal samples comparing the male peak EAE group with the male CFA+PTX group. Positive log_2_ fold changes indicate genera enriched in the male peak EAE group, while negative values indicate enrichment in the male CFA+PTX group. Only genera with a false discovery rate (FDR) < 0.05 are shown. Male peak EAE versus male sham RNA-sequencing analysis, GO (F) and KEGG (G). Only significant enrichment, FDR < 0.05, is displayed. The shape of the point indicates regulation (triangle: upregulation; circle: downregulation), the point size corresponds to gene count, and the point color indicates the FDR value. Sample sizes: male CFA+PTX (*n* = 7); male peak EAE (*n* = 9).

At the peak of EAE for each respective sex, colon fecal samples and adjacent colon tissue were collected from sham mice (females/males day 17/19) to evaluate the effects of CFA+PTX compared to EAE. Sham-treated and peak EAE males had significant differences in the Simpson and Shannon Indices (Fig. 5A/B/D), but no significance in the F/B ratio or Chao1 Index (Fig. 5C). Sham-treated males also reported significant differences compared to EAE induced males on D0 in F/B ratio and Simpson Index (Supp. Fig. 2A). Compared to sham-treated males, peak EAE males had increased relative abundance of the genus *Eisenbergiella* and Escherichia-Shigella (Fig. 5E); with the ileum of MS patients having increased relative abundance compared to healthy controls [17]. Males at peak EAE also had increased relative abundance of Runococcaceae_unclassified, *Anaerostipes*, and Christensenellaceae_unclassified compared to sham-treated males (Fig. 5E). Comparatively, sham-treated males had increased relative abundance of the genus *Alistipes, Faecalibaculum, Bacteroidetes*, and *Enterorhabdus* compared to peak EAE males (Fig. 5E).

RNA-seq analysis revealed that compared to sham treated males, peak EAE males had upregulation of genes associated with extracellular region and space, and downregulation of genes associated with defense response to virus (Fig. 5F). KEGG analysis showed that peak EAE males had upregulation of genes associated with the diseases African trypanosomiasis and Malaria compared to sham treated males (Fig. 5F). Peak EAE males also showed downregulation of genes associated with the diseases Rheumatoid arthritis, Measles, Hepatitis C, and Influenza A compared to sham treated males (Fig. 5F). Interestingly, males at the peak of EAE had upregulation of genes associated with pathways for ECM-receptor interaction, focal adhesion, and PI3K-Akt signaling pathway (Fig. 5F).

### 3.6. Comparison between EAE and CFA+PTX treatments in female mice

**Fig. 6.**
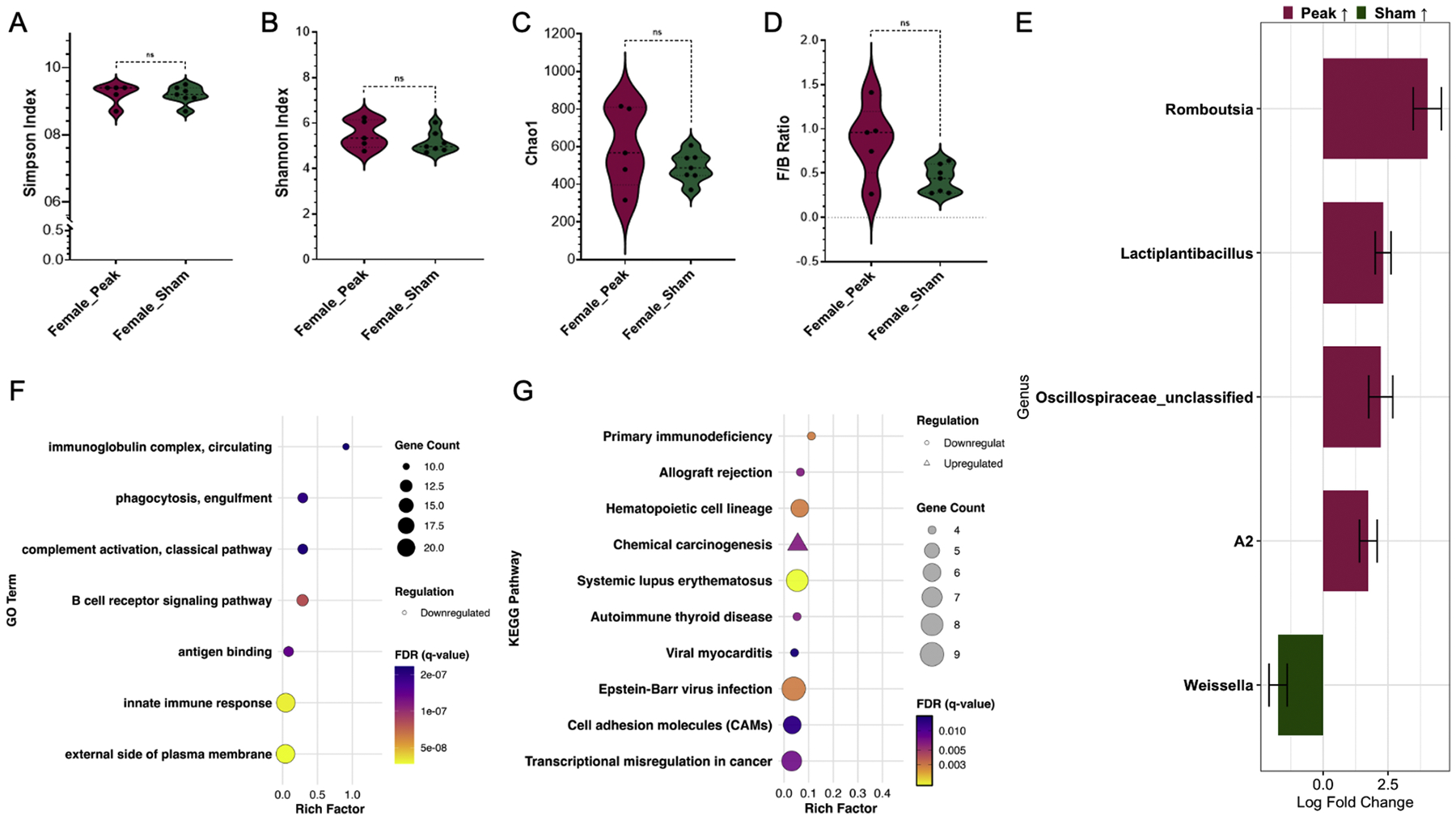
Comparison of the female colon microbiome and RNA-seq of an adjacent colon section at peak EAE versus female CFA+PTX. A) Simpson Index; B) Shannon Index; C) Chao1 Index; D) Ratio of Firmicutes to Bacteroidetes in colon fecal samples comparing female peak EAE with female CFA+PTX group; Mann-Whitney test: *, *p* < 0.05; **, *p* < 0.01; ***, *p* < 0.001. E) Bacterial genera with differential abundance in colon fecal samples comparing female peak EAE to female CFA+PTX group. Positive log_2_ fold changes indicate genera enriched in the female peak EAE group; negative values indicate enrichment in the female CFA+PTX group. Only genera with FDR < 0.05 are shown. Female peak EAE versus female sham RNA-sequencing analysis: GO (F) and KEGG (G). Only significant enrichment with FDR < 0.05 is displayed. The shape of the point indicates regulation (triangle: upregulation; circle: downregulation), the size of the point reflects gene count, and the color represents the FDR value. Sample sizes: Female CFA+PTX (*n* = 7); female peak EAE (*n* = 9).

We then investigated transcriptomic and colon microbiome changes in female mice at peak EAE compared with sham-treated mice. No significant difference was observed in Simpson, Shannon, Chao1, or F/B ratio for sham-treated vs. peak EAE in female mice (Fig. 6A–D). At the peak of EAE, female mice had increased relative abundance of the genus *Romboutsia, Lactiplantibacillus*, Oscillospiraceae_unclassified, and A2 compared to sham-treated females (Fig. 6E). Sham-treated mice had increased relative abundance of the genus *Weissella* compared to peak EAE females (Fig. 6C).

We found that peak EAE females (compared to sham-treated females) had downregulation of genes associated with the pathways for immunoglobulin complex (circulating), phagocytosis (engulfment), complement activation (classical pathway), and B cell receptor signaling pathway (Fig. 6F). Additionally, downregulation of pathways associated with primary immunodeficiency, hematopoietic cell lineage, systemic lupus erythematosus, and autoimmune thyroid disease was also reported for females at peak EAE compared to sham-treated (Fig. 6G). This trend continued with viral-associated pathways in peak EAE females; downregulation of viral myocarditis and Epstein-Barr virus infection was also seen compared to sham-treated females (Fig. 6G). Differentially expressed genes between sham-treated and peak EAE for males and females, respectively, are shown in Table 2. Overall, in male and female mice, CFA+PTX treatment altered the colon microbiome and colonic transcriptomic profile compared to EAE-induced mice at disease peak, with sex-specific differences in the colonic microenvironment in the context of CFA+PTX compared to the peak of EAE.

### 3.7. Concatenated visualization of transcriptomics and microbiome profiles during EAE progression provides sex-dependent sample clustering

**Fig. 7.**
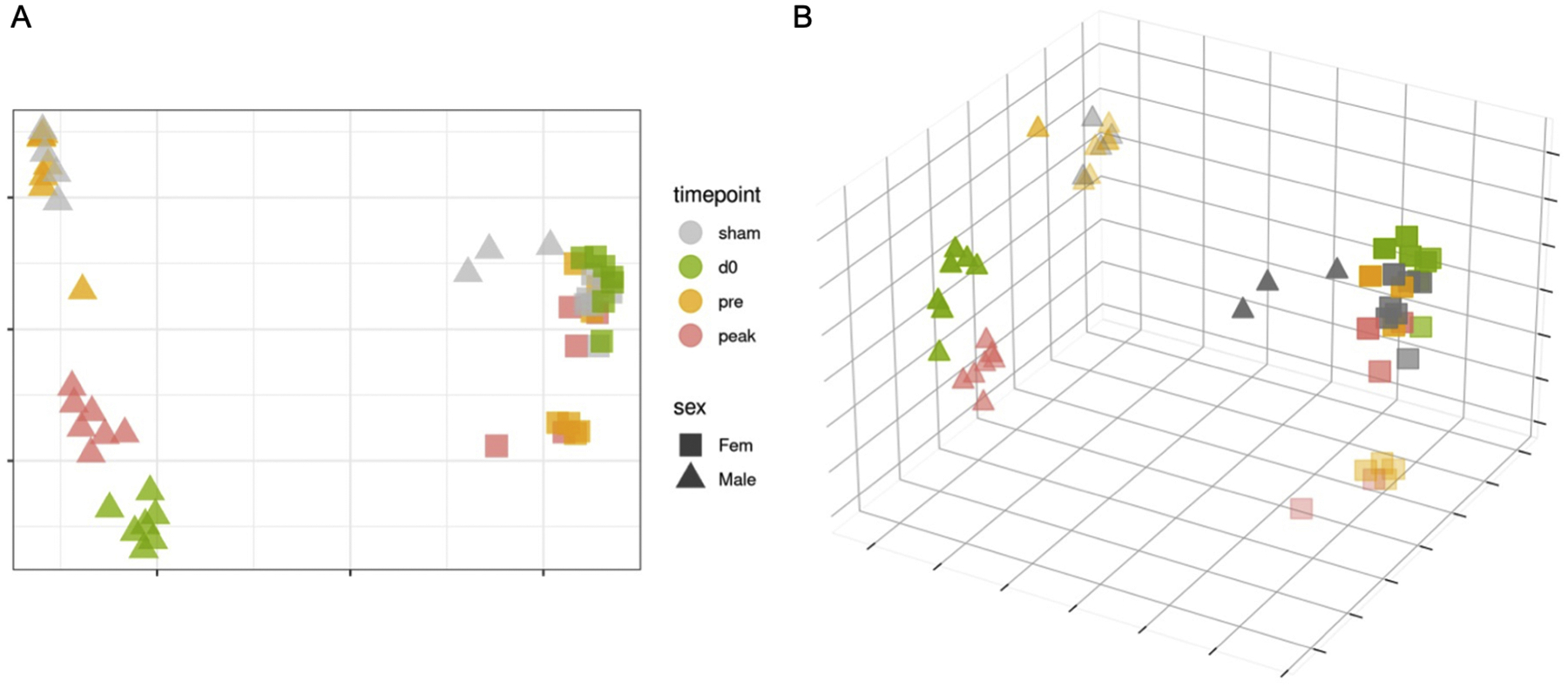
Combined visualization of the colon microbiome and colon RNA-seq for males and females at D0, pre-onset EAE, peak EAE, and CFA+PTX treatment. Concatenated colon RNA and microbiome DNA sequencing data in 2D (A) and 3D (B) using PHATE reduction. Male samples are represented by squares and female samples by triangles; colors correspond to the day of tissue collection: sham (gray), D0 (green), pre-onset EAE (orange), and peak EAE (red). In 3D plots, more transparent points indicate that the data point is farther from the viewer, reflecting the differences in microbiome and transcriptomic profiles. Supplementary Fig. 5 shows PHATE reductions for colon RNA-seq in 2D (A) and 3D (B), as well as for 16S rDNA microbiome sequencing in 2D (C) and 3D (D), each independent of the others. Sample sizes: female CFA+PTX (*n* = 7); female naïve (pre-induction of disease; D0, *n* = 7); female pre-onset EAE (*n* = 7); female peak EAE (*n* = 9); male CFA+PTX (*n* = 7); male naïve (pre-induction of disease; D0, *n* = 7); male pre-onset EAE (*n* = 7); male peak EAE (*n* = 9).

Finally, we aimed to simplify the visualization of colon profiles by combining RNA-seq and microbiome sequencing analysis (Fig. 7A/B and Supp. Fig. 4A–D). The points in Fig. 7A/B are as follows: Male samples are represented by squares and female samples by triangles; Colors correspond to the day of tissue collection: sham (gray), d0 (green), pre-onset EAE (orange), and peak EAE (red). In 3-D plots, more transparent points indicate the data point is farther from the viewer, corresponding to the compositional differences in microbiome/transcriptomic profiles. Male and female concatenated profiles were distinctly separated at pre-induction baseline (D0) when the colon-RNA-seq and microbiome data were concatenated (Fig. 7A/B). We also observed a separation between females at pre-induction (D0) and at the disease peak (Fig. 7A/B). In males, we observed a less distinct separation between time points in the concatenated RNA-seq and microbiome analyses, with the only clear distinction being pre-onset EAE compared to other male time points (Fig. 7A/B). Supplementary Fig. 5 shows PHATE reductions for colon RNA-seq and 16S rDNA microbiome sequencing in 2D and 3D, each independent of the others.

Our concatenated analysis revealed spatial similarity between females at pre-onset EAE and sham females (note that sham groups were collected at the disease peak of their respective sex) (Fig. 7A/B), indicating that sham treatment in females induces colonic microbiome and transcriptomic changes comparable to those in females at pre-onset EAE. The relative abundance analysis revealed that pre-onset EAE female mice had increased relative abundance of *Lactobacillus* and *Romboutsia*, while sham-treated female mice had increased relative abundance of *Clostridium sensu stricto 1, Monoglobus*, and *Colidextribacter* (Supplementary Fig. 6). RNA-seq KEGG analysis revealed pre-onset EAE females had reduced RF for genes associated with primary immunodeficiency, innate immune response, cell adhesion molecules (CAMs), and the external side of the plasma membrane (Supplementary Fig. 7). Overall, we found that when we concatenated our colon RNA-seq and colon fecal samples and used PHATE analysis, females had more defined clusters at each time point compared to males throughout EAE progression.

## 4. Discussion

Active EAE is the most widely used animal model for studying CNS demyelinating inflammatory diseases, such as MS, and is fundamental to understanding the disease’s immunopathology and developing immune-modifying therapies against MS. More recently, many laboratories, including ours, have used EAE as an experimental model to study the bidirectional relationship between gut microbes and the CNS in the context of autoimmunity [1,54]. In this study, we conducted a descriptive analysis of the effects of EAE induction and the adjuvants used in the model on the colon microbiome and transcriptomics profiles of male and female mice throughout disease progression. Overall, we observed fundamental differences at baseline; however, as EAE progressed, these changes became less pronounced at the disease peak.

The administration of CFA and PTX is well known to induce inflammation [55–57]. CFA injection has been shown to partially alter the intestinal immune response, increasing serum levels of IgA and IgG, enhancing the production of inflammatory cytokines IL-17 and IFN-γ, and promoting Th1/17 differentiation [58,59]. We found that at the peak of EAE, males showed upregulation of T helper 17 cell chemotaxis compared to females (Fig. 4F). Previous work has also shown that CFA/EAE induction alters the microbiome in secreted fecal samples from female mice [46,59,60]. Finding that the relative abundance of *Lactobacillus* decreases, while the relative abundance of *Romboutsia* and various Clostridia populations increases as EAE progresses [60]. Additionally, they found that CFA treatment alone induced similar changes to secreted fecal pellets, albeit to a lesser extent than EAE-induced mice [60]. Overall, our findings indicate that CFA+PTX treatment induces changes in colon transcriptomics and the colon microbiome, and we and others have elucidated that this confounding variable warrants further investigation in the context of animal autoimmune disease models [46, 59,61].

The role of biological sex, the composition of the gut microbiome, and its relationship to inflammation are also topics of growing interest [10,62,63]. It has been established that the gut microbiome of male and female mice differs, with males having lower microbial diversity and richness compared to females [64,65], with our findings showing males having significant changes in the colon microbiome throughout EAE progression, but not females (Fig. 2A). The microbiome of the ileum in MS patients can induce EAE in transgenic TCR (MOG_96–105_) mice, with a higher incidence in females compared to males, and *Lachnospiraceae* being the likely inducer [17]. Our findings show that at pre-induction baseline, males had a higher relative abundance of *Lachnospiraceae* than females, and females had reduced relative abundance at pre-onset EAE compared to D0 (Fig. 2E and 3E). Female germ-free mice that received a male fecal microbiome transfer had increased intestinal inflammation and weight loss [65]. Furthermore, testosterone induces interleukin-33 (IL-33), contributing to protection in male EAE-SJL mice by promoting a Th2 response rather than a Th1/17 response [63]. IL-33 is essential for innate immune cell activity and is protective in EAE; however, conflicting findings exist regarding its role in intestinal inflammation [66–68]. Interestingly, males exhibit a more robust inflammatory response when it is induced with EAE or treated with CFA+PTX; however, this is mitigated by higher baseline percentages of anti-inflammatory and regulatory adaptive and innate immune cells in the spleen and spinal cord [62].

The concatenated space between pre-onset females and shams does not necessarily indicate a sudden loss of microbial diversity. Instead, it might indicate a shared gut microenvironmental shift triggered by adjuvant immunization with the active EAE adjuvants (CFA+PTX) before CNS-specific pathology takes over at the severe stages of the disease. The specific differences in bacterial taxa between pre-onset and sham (CFA+PTX) female mice could actively drive the observed clustering, a possibility we are currently evaluating. Published works suggest that specific bacteria could influence an immunological shift that skews the host toward an inflammatory, encephalitogenic stage [69–71].

Our study has several limitations: 1) A limited number of samples per group were analyzed (*n* = 7 for sham treatment, pre-induction, and pre-onset EAE, and *n* = 9 for peak EAE for each sex). Larger, more powerful comparisons will establish whether the pathways and microbial profiles observed in our study are translatable to larger populations; 2) Our study only included samples isolated from a single section of the colon and not sections of the small intestine. There are known differences in gut microbiome biodistribution and in the chemical and physiological characteristics across sections of the gastrointestinal tract [72,73]. Because of that, follow-up microbiome sequencing, transcriptomics, and subsequent concatenated studies are worth performing; 3) Our microbial analysis only included bacterial/archaeal 16S rDNA sequencing, and did not consider the potential effects of sex and disease progression on the mycobiome, observed to be impacted in MS individuals [74,75] and the EAE model [76]; 4) Technological advances allow us to discern the cell type-specific transcriptome by single cell RNA-seq (scRNA-seq), which was not performed in our study; Importantly, 5) our study compared samples from different mice for analyzing specific timepoints within EAE progression, which adds potential interindividual variability; 6) EAE mice experience significant weight loss as disease progresses as a result of reduced eating and drinking (Fig. 1C). Although we ease access to food and water ad libitum to all EAE mice, the reduction in caloric and water intake affects significantly the physiology of the gut, which could result in reduced fecal content and altered microbiome composition, constituting a cofounding factor for transcriptomics and microbiome analyses. An increasing body of evidence indicates that constipation is present in the EAE model and in MS patients, with recent reports highlighting that reducing anaerobic microbes induces constipation in taxa associated with MS and the EAE model [77,78]. It has also been reported that changes in the microbiome composition can alter metabolite and neurotransmitter production, hence, altering intestinal motility [79,80]. As a result, differences in profiles between sexes could become less relevant as the disease progresses due to fasting. Thus, fasting is an inherent characteristic of the model and should be acknowledged; 7) Active EAE requires CFA+PTX administration. We cannot rule out that the differences in omics profiles observed between the CFA+PTX controls and EAE mice, particularly at the peak of the disease, are due to differences in intake levels. Intermittent fasting before symptom onset attenuates disease severity in EAE mice [81,82]. In addition, it was shown that mice given ~30 % of their average caloric intake had reduced EAE severity compared with the regular-diet EAE group [82]. A future, more rigorous approach will incorporate a CFA/PTX control group with a diet restricted to the intake volume consumed by EAE groups; Finally, 8) the study is entirely descriptive and does not recapitulate CNS neuroinflammation and demyelination, which have been described extensively in active EAE [83–85]. For instance, we recently published the effects of active EAE induction on the transcriptomics profile of EAE mice [23].

In conclusion, we present a preliminary analysis of the proximal colon, distinct from other studies of the microbiome based on stool samples and immunological studies of the small intestine in EAE mice, and offer a new perspective on the effects of active EAE on the gut microbiome and colonic transcriptomics profiles. We observed that the colonic microenvironment is sexually dimorphic in C57BL/6 J mice before EAE induction. As EAE progresses, the differences in colon transcriptomics and microbiome become less apparent. The study presents a novel perspective on sex dimorphism of disease in the colon’s microenvironment. Future mechanistic studies will ascertain the biological relevance of the interactions between colonic microbes and the host’s mucosal barriers.

## Supplementary Material

MMC6

MMC7

MMC5

MMC4

MMC3

MMC1

MMC2

Supplementary material associated with this article can be found, in the online version, at doi:10.1016/j.clicom.2026.03.003.

## Figures and Tables

**Table 1:** Top 10 differentially expressed genes in the colon, comparing males and females at day 0, pre-onset, and disease peak ranked by FDR value*.

Comparison (male vs Female)	Gene Symbol	Regulation	log2FC	FDR value	*p*-value
**Day 0**	Eif2s3y	Up	17.57	<1e-300	<1e-300
	Ddx3y	Up	17	<1e-300	<1e-300
	Kdm5d	Up	14.68	<1e-300	<1e-300
	Uty	Up	14.95	<1e-300	<1e-300
	Gm28588	Up	16.08	<1e-300	<1e-300
	Gm29650	Up	14.98	<1e-300	<1e-300
	Gm2098	Up	11.6	<1e-300	<1e-300
	Rbm3	Up	1.06	<1e-300	<1e-300
	Xist	Down	−10.1	<1e-300	<1e-300
	Kdm6a	Down	−1.08	<1e-300	<1e-300
**Pre-onset**	Eif2s3y	Up	17.2	<1e-300	<1e-300
	Ddx3y	Up	16.22	<1e-300	<1e-300
	Kdm5d	Up	14.52	<1e-300	<1e-300
	Uty	Up	14.79	<1e-300	<1e-300
	Gm28588	Up	16.69	<1e-300	<1e-300
	Gm29650	Up	14.35	<1e-300	<1e-300
	Gm2098	Up	11.87	<1e-300	<1e-300
	Xist	Down	−11.3	<1e-300	<1e-300
	Kdm6a	Down	−1.07	<1e-300	<1e-300
	Sec14l4	Down	−2.4	<1e-300	<1e-300
**Peak**	Ddx3y	Up	12.32	<1e-300	<1e-300
	Eif2s3y	Up	17.07	<1e-300	<1e-300
	Kdm5d	Up	14.6	<1e-300	<1e-300
	Uty	Up	14.91	<1e-300	<1e-300
	Gm28588	Up	16.1	<1e-300	<1e-300
	Gm49909	Up	16.82	<1e-300	<1e-300
	Gm29650	Up	13.49	<1e-300	<1e-300
	Gm45062	Up	12.33	<1e-300	<1e-300
	Ugt1a9	Up	13.16	<1e-300	<1e-300
	Xist	Down	−13.26	<1e-300	<1e-300

* Only significant genes are shown (FDR < 0.05, |log2FC| > 1). Positive fold change indicates increased expression in males (upregulation - Up) and negative fold change indicates increased expression in females (downregulation - Down).

**Table 2:** Top 10 differentially expressed genes in the colon, comparing CFA+PTX (Sham) and peak EAE in males and females, respectively.

Comparison (Peak vs Sham)	Gene Symbol	Regulation	log2FC	FDR value	*p*-value
**Female**	Ces1d	Up	1.46	<1e-300	<1e-300
	Gm20458	Up	12.52	<1e-300	<1e-300
	Dclre1a	Down	−1.2	<1e-300	<1e-300
	Crybb3	Down	−1.7	<1e-300	<1e-300
	Als2	Down	−1.08	<1e-300	<1e-300
	Rdh16f1	Down	−12.82	<1e-300	<1e-300
	Gm20521	Down	−12.87	<1e-300	<1e-300
	Gm49909	Down	−16.95	<1e-300	<1e-300
	Ifi27l2a	Down	−1.44	<1e-300	<1e-300
	Ighv7–3	Down	−3.25	<1e-300	<1e-300
**Male**	Tppp	Up	1.41	<1e-300	<1e-300
	Cdcp3	Up	10.52	<1e-300	<1e-300
	Gm43738	Up	12.25	<1e-300	<1e-300
	Oas2	Down	−3.29	<1e-300	<1e-300
	Rtp4	Down	−2.5	<1e-300	<1e-300
	Ciart	Down	−1.27	<1e-300	<1e-300
	Fbxl12os	Down	−1.12	<1e-300	<1e-300
	Gm4779	Down	−12.03	<1e-300	<1e-300
	Afm	Down	−1.1	<1e-300	<1e-300
	Gm20431	Down	−10.8	<1e-300	<1e-300

* Only significant genes are shown (FDR 〈 0.05, |log2FC| 〉 1), with genes ranked by FDR value. Positive fold change indicates increased expression in the peak EAE group (upregulation - Up) and negative fold change indicates increased expression in CFA+PTX group (downregulation - Down).

## Data Availability

The data discussed in this publication are accessible through the GEO repository number GSE289817.

## References

[1] Schumacher SM, Doyle WJ, Hill K, Ochoa-Repáraz J, Gut microbiota in multiple sclerosis and animal models, FEBS J. (2024), 10.1111/febs.17161. PMC11607183 38817090

[2] Diakou I, Papakonstantinou E, Papageorgiou L, Pierouli K, Dragoumani K, Spandidos D, Bacopoulou F, Chrousos G, Goulielmos G, Eliopoulos E, Vlachakis D, Multiple sclerosis and computational biology (Review), Biomed. Rep 17 (2022) 96, 10.3892/br.2022.1579. 36382258 PMC9634047

[3] Ysrraelit MC, Correale J, Impact of sex hormones on immune function and multiple sclerosis development, Immunology 156 (2019) 9–22, 10.1111/imm.13004. 30222193 PMC6283654

[4] Thompson AJ, Baranzini SE, Geurts J, Hemmer B, Ciccarelli O, Multiple sclerosis, Lancet 391 (2018) 1622–1636, 10.1016/S0140-6736(18)30481-1. 29576504

[5] Walton C, King R, Rechtman L, Kaye W, Leray E, Marrie RA, Robertson N, La Rocca N, Uitdehaag B, Van Der Mei I, Wallin M, Helme A, Angood Napier C, Rijke N, Baneke P, Rising prevalence of multiple sclerosis worldwide: insights from the Atlas of MS, third edition, Mult. Scler 26 (2020) 1816–1821, 10.1177/1352458520970841. 33174475 PMC7720355

[6] Wallin MT, Culpepper WJ, Campbell JD, Nelson LM, Langer-Gould A, Marrie RA, Cutter GR, Kaye WE, Wagner L, Tremlett H, Buka SL, Dilokthornsakul P, Topol B, Chen LH, LaRocca NG, The prevalence of MS in the United States: a population-based estimate using health claims data, Neurology. 92 (2019) e1029–e1040, 10.1212/WNL.0000000000007035. 30770430 PMC6442006

[7] Sandi D, Fricska-Nagy Z, Bencsik K, Vécsei L, Neurodegeneration in multiple sclerosis: symptoms of silent progression, biomarkers and neuroprotective therapy—Kynurenines are important players, Molecules 26 (2021) 3423, 10.3390/molecules26113423. 34198750 PMC8201043

[8] Ford H, Clinical presentation and diagnosis of multiple sclerosis, Clin Med 20 (2020) 380–383, 10.7861/clinmed.2020-0292. PMC7385797 32675142

[9] Steinman L, Immunology of relapse and remission in multiple sclerosis, Annu. Rev. Immunol 32 (2014) 257–281, 10.1146/annurev-immunol-032713-120227. 24438352

[10] Kim YS, Unno T, Kim B-Y, Park M-S, Sex differences in gut microbiota, World J. Mens. Health 38 (2020) 48, 10.5534/wjmh.190009. 30929328 PMC6920072

[11] Zhou X, Baumann R, Gao X, Mendoza M, Singh S, Katz Sand I, Xia Z, Cox LM, Chitnis T, Yoon H, Moles L, Caillier SJ, Santaniello A, Ackermann G, Harroud A, Lincoln R, Gomez R, Gonźalez Peña A, Digga E, Hakim DJ, Vazquez-Baeza Y, Soman K, Warto S, Humphrey G, Farez M, Gerdes LA, Oksenberg JR, Zamvil SS, Chandran S, Connick P, Otaegui D, Castillo-Triviño T, Hauser SL, Gelfand JM, Weiner HL, Hohlfeld R, Wekerle H, Graves J, Bar-Or A, Cree BAC, Correale J, Knight R, Baranzini SE, Gut microbiome of multiple sclerosis patients and paired household healthy controls reveal associations with disease risk and course, Cell 185 (2022), 10.1016/j.cell.2022.08.021, 3467–3486.e16. 36113426 PMC10143502

[12] Hoffman K, Brownell Z, Doyle WJ, Ochoa-Repáraz J, The immunomodulatory roles of the gut microbiome in autoimmune diseases of the central nervous system: multiple sclerosis as a model, J. Autoimmun 137 (2023) 102957, 10.1016/j.jaut.2022.102957. 36435700 PMC10203067

[13] Saresella M, Mendozzi L, Rossi V, Mazzali F, Piancone F, LaRosa F, Marventano I, Caputo D, Felis GE, Clerici M, Immunological and clinical effect of diet modulation of the gut microbiome in multiple sclerosis patients: a pilot study, Front. Immunol 8 (2017) 1391, 10.3389/fimmu.2017.01391. 29118761 PMC5661395

[14] Colpitts SL, Kasper EJ, Keever A, Liljenberg C, Kirby T, Magori K, Kasper LH, Ochoa-Reparaz J, A bidirectional association between the gut microbiota and CNS disease in a biphasic murine model of multiple sclerosis, Gut. Microbes 8 (2017) 561–573. 28708466 10.1080/19490976.2017.1353843 PMC5730387

[15] Sell LB, Ramelow CC, Kohl HM, Hoffman K, Bains JK, Doyle WJ, Strawn KD, Hevrin T, Kirby TO, Gibson KM, Roullet J-B, Ochoa-Repáraz J, Farnesol induces protection against murine CNS inflammatory demyelination and modifies gut microbiome, Clin. Immunol (2021) 108766, 10.1016/j.clim.2021.108766. 34091018 PMC8660955

[16] Daberkow DP, Hoffman K, Kohl HM, Long T, Kirby TO, Ochoa-Repáraz J, Microbiome methods in experimental autoimmune encephalomyelitis, Curr. Protoc 1 (2021), 10.1002/cpz1.314. PMC9540342 34870901

[17] Yoon H, Gerdes LA, Beigel F, Sun Y, Kövilein J, Wang J, Kuhlmann T, Flierl-Hecht A, Haller D, Hohlfeld R, Baranzini SE, Wekerle H, Peters A, Multiple sclerosis and gut microbiota: lachnospiraceae from the ileum of MS twins trigger MS-like disease in germfree transgenic mice—An unbiased functional study, Proc. Natl. Acad. Sci. U.S.A 122 (2025), 10.1073/pnas.2419689122. PMC12067282 40258140

[18] Camara-Lemarroy CR, Metz L, Meddings JB, Sharkey KA, Wee Yong V, The intestinal barrier in multiple sclerosis: implications for pathophysiology and therapeutics, Brain 141 (2018) 1900–1916, 10.1093/brain/awy131. 29860380 PMC6022557

[19] Camara-Lemarroy CR, Metz LM, Yong VW, Focus on the gut-brain axis: multiple sclerosis, the intestinal barrier and the microbiome, WJG 24 (2018) 4217–4223, 10.3748/wjg.v24.i37.4217. 30310254 PMC6175760

[20] Tang Q, Jin G, Wang G, Liu T, Liu X, Wang B, Cao H, Current sampling methods for gut microbiota: a call for more precise devices, Front. Cell Infect. Microbiol 10 (2020) 151, 10.3389/fcimb.2020.00151. 32328469 PMC7161087

[21] Tap J, Derrien M, Törnblom H, Brazeilles R, Cools-Portier S, Doŕe J, Störsrud S, Le Nevé B, Öhman L, Simŕen M, Identification of an intestinal microbiota signature associated with severity of irritable bowel syndrome, Gastroenterology 152 (2017), 10.1053/j.gastro.2016.09.049, 111–123.e8. 27725146

[22] Rezaei Nejad H, Oliveira BCM, Sadeqi A, Dehkharghani A, Kondova I, Langermans JAM, Guasto JS, Tzipori S, Widmer G, Sonkusale SR, Ingestible osmotic pill for In vivo sampling of gut microbiomes, Adv. Intell. Syst 1 (2019) 1900053, 10.1002/aisy.201900053.

[23] Doyle WJ, Walters D, Shi X, Hoffman K, Magori K, Roullet J-B, Ochoa-Repáraz J, Farnesol brain transcriptomics in CNS inflammatory demyelination, Clin. Immunol 255 (2023) 109752, 10.1016/j.clim.2023.109752. 37673223 PMC10619994

[24] Mangalam A, Shahi SK, Luckey D, Karau M, Marietta E, Luo N, Choung RS, Ju J, Sompallae R, Gibson-Corley K, Patel R, Rodriguez M, David C, Taneja V, Murray J, Human gut-derived commensal bacteria suppress CNS inflammatory and demyelinating disease, CellReports 20 (2017) 1269–1277. 10.1016/j.celrep.2017.07.031 PMC5763484 28793252

[25] Ladakis DC, Bhargava P, The role of gut dysbiosis and potential approaches to target the gut microbiota in multiple sclerosis, CNS Drugs 37 (2023) 117–132, 10.1007/s40263-023-00986-w. 36690786

[26] Ivanov II, de L. Frutos R, Manel N, Yoshinaga K, Rifkin B, Sartor RB, Finlay BB, Littman DR, Specific microbiota direct the differentiation of Th17 cells in the mucosa of the small intestine, (2009). 10.1016/j.chom.2008.09.009 PMC2597589 18854238

[27] Khoshbin K, Khanna L, Maselli D, Atieh J, Breen-Lyles M, Arndt K, Rhoten D, Dyer RB, Singh RJ, Nayar S, Bjerkness S, Harmsen WS, Busciglio I, Camilleri M, Development and validation of test for “leaky gut” small intestinal and colonic permeability using sugars in healthy adults, Gastroenterology 161 (2021), 10.1053/j.gastro.2021.04.020, 463–475.e13. 33865841 PMC8328885

[28] Muto H, Honda T, Tanaka T, Yokoyama S, Yamamoto K, Ito T, Imai N, Ishizu Y, Maeda K, Ishikawa T, Adachi S, Sato C, Tsuji NM, Ishigami M, Fujishiro M, Kawashima H, Proteomic analysis reveals changes in tight junctions in the small intestinal epithelium of mice fed a high-fat diet, Nutrients. 15 (2023) 1473, 10.3390/nu15061473. 36986203 PMC10056729

[29] Dedoni S, Scherma M, Camoglio C, Siddi C, Dazzi L, Puliga R, Frau J, Cocco E, Fadda P, An overall view of the most common experimental models for multiple sclerosis, Neurobiol. Dis 184 (2023) 106230, 10.1016/j.nbd.2023.106230. 37453561

[30] Martin M, Cutadapt removes adapter sequences from high-throughput sequencing reads, EMBnet.J 17 (2011) 10–12, 10.14806/ej.17.1.200.

[31] Kim D, Langmead B, Salzberg SL, HISAT: a fast spliced aligner with low memory requirements, Nat. Methods 12 (2015) 357–360, 10.1038/nmeth.3317. 25751142 PMC4655817

[32] Pertea M, Pertea GM, Antonescu CM, Chang T-C, Mendell JT, Salzberg SL, StringTie enables improved reconstruction of a transcriptome from RNA-seq reads, Nat. Biotechnol 33 (2015) 290–295, 10.1038/nbt.3122. 25690850 PMC4643835

[33] Robinson MD, McCarthy DJ, Smyth GK, edgeR: a bioconductor package for differential expression analysis of digital gene expression data, Bioinformatics 26 (2010) 139–140, 10.1093/bioinformatics/btp616. 19910308 PMC2796818

[34] Love MI, Huber W, Anders S, Moderated estimation of fold change and dispersion for RNA-seq data with DESeq2, Genome Biol. 15 (2014) 550, 10.1186/s13059-014-0550-8. 25516281 PMC4302049

[35] Xie Y (2025). knitr: A General-Purpose Package for Dynamic Report Generation in R. R package version 1.51, https://yihui.org/knitr/.

[36] Yu G, Wang L-G, Han Y, He Q-Y, clusterProfiler: an R package for comparing biological themes among gene clusters, OMICS: J. Integr. Biol 16 (2012) 284–287, 10.1089/omi.2011.0118. PMC3339379 22455463

[37] Yu G, enrichplot: visualization of Functional enrichment result, (n.d.).

[38] Callahan BJ, McMurdie PJ, Rosen MJ, Han AW, Johnson AJA, Holmes SP, DADA2: high-resolution sample inference from Illumina amplicon data, Nat. Methods 13 (2016) 581–583, 10.1038/nmeth.3869. 27214047 PMC4927377

[39] McGinnis S, Madden TL, BLAST: at the core of a powerful and diverse set of sequence analysis tools, Nucleic. Acids. Res 32 (2004) W20–W25, 10.1093/nar/gkh435. 15215342 PMC441573

[40] Lin H, Peddada SD, Multigroup analysis of compositions of microbiomes with covariate adjustments and repeated measures, Nat. Methods 21 (2024) 83–91, 10.1038/s41592-023-02092-7. 38158428 PMC10776411

[41] McMurdie PJ, Holmes S, phyloseq: an R package for reproducible interactive analysis and graphics of microbiome census data, PLoS One 8 (2013) e61217. 23630581 10.1371/journal.pone.0061217 PMC3632530

[42] Gómez-Rubio V, ggplot2 - Elegant graphics for Data analysis (2nd Edition), J. Stat. Soft (2017) 77, 10.18637/jss.v077.b02.

[43] Benjamini Y, Hochberg Y, Controlling the false discovery rate: a practical and powerful approach to multiple testing, J. R. Stat. Soc. B: Stat. Methodol 57 (1995) 289–300, 10.1111/j.2517-6161.1995.tb02031.x.

[44] Moon KR, van Dijk D, Wang Z, Gigante S, Burkhardt DB, Chen WS, Yim K, van den Elzen A, Hirn MJ, Coifman RR, Ivanova NB, Wolf G, Krishnaswamy S, Visualizing structure and transitions in high-dimensional biological data, Nat. Biotechnol 37 (2019) 1482–1492, 10.1038/s41587-019-0336-3. 31796933 PMC7073148

[45] Sun Y-J, Zhang Q-Y, Liu F, Chen L, Wang J-F, Polysaccharides isolated from Cibotium barometz attenuate chronic inflammatory pain: molecular chemical structure and role of phenylalanine, Int. J. Biol. Macromol 297 (2025) 139911, 10.1016/j.ijbiomac.2025.139911. 39818377

[46] Lazarevíc M, Stanisavljevíc S, Nikolovski N, Dimitrijevíc M, Miljkovíc Đ, Complete Freund’s adjuvant as a confounding factor in multiple sclerosis research, Front. Immunol 15 (2024), 10.3389/fimmu.2024.1353865. PMC10902151 38426111

[47] Kozhieva M, Naumova N, Alikina T, Boyko A, Vlassov V, Kabilov MR, The core of gut life: firmicutes profile in patients with relapsing-remitting multiple sclerosis, Life 11 (2021) 55, 10.3390/life11010055. 33466726 PMC7828771

[48] Magne F, Gotteland M, Gauthier L, Zazueta A, Pesoa S, Navarrete P, Balamurugan R, The firmicutes/bacteroidetes ratio: a relevant marker of gut dysbiosis in obese patients? Nutrients. 12 (2020) 1474, 10.3390/nu12051474. 32438689 PMC7285218

[49] Kanehisa M, Goto S, KEGG: kyoto Encyclopedia of Genes and Genomes, (n.d.) 4. 10.1093/nar/28.1.27 PMC102409 10592173

[50] The Gene Ontology Consortium, The Gene Ontology project in 2008, Nucleic. Acids. Res 36 (2008) D440–D444, 10.1093/nar/gkm883. 17984083 PMC2238979

[51] Bui TPN, Mannerås-Holm L, Puschmann R, Wu H, Troise AD, Nijsse B, Boeren S, Bäckhed F, Fiedler D, deVos WM, Conversion of dietary inositol into propionate and acetate by commensal Anaerostipes associates with host health, Nat. Commun 12 (2021) 4798, 10.1038/s41467-021-25081-w. 34376656 PMC8355322

[52] Nie K, Ma K, Luo W, Shen Z, Yang Z, Xiao M, Tong T, Yang Y, Wang X, Roseburia intestinalis: a beneficial gut organism from the discoveries in genus and species, Front. Cell Infect. Microbiol 11 (2021) 757718, 10.3389/fcimb.2021.757718. 34881193 PMC8647967

[53] Cabrera Zapata LE, Cisternas CD, Sosa C, Garcia-Segura LM, Arevalo MA, Cambiasso MJ, X-linked histone H3K27 demethylase Kdm6a regulates sexually dimorphic differentiation of hypothalamic neurons, Cell Mol. Life Sci 78 (2021) 7043–7060, 10.1007/s00018-021-03945-0. 34633482 PMC8558156

[54] Hoffman K, Doyle WJ, Schumacher SM, Ochoa-Repáraz J, Gut microbiome-modulated dietary strategies in EAE and multiple sclerosis, Front. Nutr 10 (2023) 1146748, 10.3389/fnut.2023.1146748. 37063324 PMC10090556

[55] Arimoto H, Tanuma N, Jee Y, Miyazawa T, Shima K, Matsumoto Y, Analysis of experimental autoimmune encephalomyelitis induced in F344 rats by pertussis toxin administration, J. Neuroimmunol 104 (2000) 15–21, 10.1016/S0165-5728(99)00242-8. 10683510

[56] Pastor S, Minguela A, Mi W, Ward ES, Autoantigen immunization at different sites reveals a role for anti-inflammatory effects of IFN-γ in regulating susceptibility to experimental autoimmune encephalomyelitis, J. Immunol 182 (2009) 5268–5275, 10.4049/jimmunol.0800681. 19380773 PMC2766852

[57] Murugesan N, Paul D, Lemire Y, Shrestha B, Ge S, Pachter JS, Active induction of experimental autoimmune encephalomyelitis by MOG35–55 peptide immunization is associated with differential responses in separate compartments of the choroid plexus, Fluids. Barriers. CNS 9 (2012) 15, 10.1186/2045-8118-9-15. 22870943 PMC3493354

[58] Khadka S, Omura S, Sato F, Tsunoda I, Adjuvant injections altered the ileal and fecal microbiota differently with changes in immunoglobulin isotypes and antimycobacterial antibody responses, IJMS 24 (2023) 2818, 10.3390/ijms24032818. 36769136 PMC9917480

[59] Mone K, Singh S, Abdullatif F, Sur M, Rasquinha MT, Seravalli J, Zinniel DK, Mukhopadhyay I, Barletta RG, Gebregiworgis T, Reddy J, Immunization with Complete Freund’s adjuvant reveals trained immunity-like features in A/J mice, Vaccines (Basel) 13 (2025) 768, 10.3390/vaccines13070768. 40733745 PMC12300380

[60] Johanson DM, Goertz JE, Marin IA, Costello J, Overall CC, Gaultier A, Experimental autoimmune encephalomyelitis is associated with changes of the microbiota composition in the gastrointestinal tract, Sci. Rep 10 (2020) 15183, 10.1038/s41598-020-72197-y. 32938979 PMC7494894

[61] Liu M, Li S, Cao N, Wang Q, Liu Y, Xu Q, Zhang L, Sun C, Xiao X, Yao J, Intestinal flora, intestinal metabolism, and intestinal immunity changes in complete Freud’s adjuvant-rheumatoid arthritis C57BL/6 mice, Int. Immunopharmacol 125 (2023) 111090, 10.1016/j.intimp.2023.111090. 37866312

[62] Wiedrick J, Meza-Romero R, Gerstner G, Seifert H, Chaudhary P, Headrick A, Kent G, Maestas A, Offner H, Vandenbark AA, Sex differences in EAE reveal common and distinct cellular and molecular components, Cell Immunol. 359 (2021) 104242, 10.1016/j.cellimm.2020.104242. 33190849 PMC7770093

[63] Russi AE, Ebel ME, Yang Y, Brown MA, Male-specific IL-33 expression regulates sex-dimorphic EAE susceptibility, Proc. Natl. Acad. Sci. U.S.A 115 (2018), 10.1073/pnas.1710401115. PMC5816140 29378942

[64] Gao A, Su J, Liu R, Zhao S, Li W, Xu X, Li D, Shi J, Gu B, Zhang J, Li Q, Wang X, Zhang Y, Xu Y, Lu J, Ning G, Hong J, Bi Y, Gu W, Wang J, Wang W, Sexual dimorphism in glucose metabolism is shaped by androgen-driven gut microbiome, Nat. Commun 12 (2021), 10.1038/s41467-021-27187-7. PMC8648805 34873153

[65] Fransen F, van Beek AA, Borghuis T, Meijer B, Hugenholtz F, van der Gaastde Jongh C, Savelkoul HF, de Jonge MI, Faas MM, Boekschoten MV, Smidt H, El Aidy S, de Vos P, The impact of gut microbiota on gender-specific differences in immunity, Front. Immunol 8 (2017) 754, 10.3389/fimmu.2017.00754. 28713378 PMC5491612

[66] Lee C-R, Kwak Y, Yang T, Han JH, Park S-H, Ye MB, Lee W, Sim K-Y, Kang J-A, Kim Y-C, Mazmanian SK, Park S-G, Myeloid-derived suppressor cells are controlled by regulatory T cells via TGF-β during Murine Colitis, CellReports 17 (2016) 3219–3232. 10.1016/j.celrep.2016.11.062 28009291

[67] Williams MA, O’Callaghan A, Corr SC, IL-33 and IL-18 in inflammatory bowel disease etiology and microbial interactions, Front. Immunol 10 (2019) 1091, 10.3389/fimmu.2019.01091. 31139196 PMC6527769

[68] Hung L-Y, Pastore CF, Douglas B, Herbert DR, Myeloid-derived IL-33 limits the severity of dextran sulfate sodium–Induced colitis, Am. J. Pathol 191 (2021) 266–273, 10.1016/j.ajpath.2020.11.004. 33245913 PMC7863133

[69] Caspani G, Green M, Swann JR, Foster JA, Microbe-immune crosstalk: evidence that T cells influence the development of the brain metabolome, IJMS 23 (2022) 3259, 10.3390/ijms23063259. 35328680 PMC8952415

[70] Cox LM, Weiner HL, The microbiome requires a genetically susceptible host to induce central nervous system autoimmunity, Proc. Natl. Acad. Sci. U.S.A 117 (2020) 27764–27766, 10.1073/pnas.2020106117. 33122445 PMC7668160

[71] Ren S, Zhang X, Guan H, Wu L, Yu M, Hou D, Yan Y, Fang X, Lactobacillus acidipiscis induced regulatory Gamma Delta T cells and attenuated experimental autoimmune encephalomyelitis, Front. Immunol 12 (2021) 623451, 10.3389/fimmu.2021.623451. 33679767 PMC7933195

[72] Mann ER, Bernardo D, English NR, Landy J, Al-Hassi HO, Peake ST, Man R, Elliott TR, Spranger H, Lee GH, Parian A, Brant SR, Lazarev M, Hart AL, Li X, Knight SC, Compartment-specific immunity in the human gut: properties and functions of dendritic cells in the colon versus the ileum, Gut 65 (2016) 256–270, 10.1136/gutjnl-2014-307916. 25666191 PMC4530083

[73] Belkaid Y, Naik S, Compartmentalized and systemic control of tissue immunity by commensals, Nat. Immunol 14 (2013) 646–653. 23778791 10.1038/ni.2604 PMC3845005

[74] Shah S, Locca A, Dorsett Y, Cantoni C, Ghezzi L, Lin Q, Bokoliya S, Panier H, Suther C, Gormley M, Liu Y, Evans E, Mikesell R, Obert K, Salter A, Cross AH, Tarr PI, Lovett-Racke A, Piccio L, Zhou Y, Alterations of the gut mycobiome in patients with MS, EBioMedicine 71 (2021) 103557, 10.1016/j.ebiom.2021.103557. 34455391 PMC8399064

[75] Yadav M, Ali S, Shrode RL, Shahi SK, Jensen SN, Hoang J, Cassidy S, Olalde H, Guseva N, Paullus M, Cherwin C, Wang K, Cho T, Kamholz J, Mangalam AK, Multiple sclerosis patients have an altered gut mycobiome and increased fungal to bacterial richness, PLoS One 17 (2022) e0264556, 10.1371/journal.pone.0264556. 35472144 PMC9041819

[76] Hill K, LaFollette A, Kirby TO, Negrete S, Babcock D, Felton K, Kohl H, Sharma K, Castillo A, Roullet J, Gibson KM, Ochoa-Repáraz J, Impact of a GABA -producing LACTOCOCCUS LACTIS on microbiota and mycobiota during CNS inflammatory demyelination, FASEB Bioadv. 8 (2026) e70085, 10.1096/fba.2025-00082. 41509630 PMC12777695

[77] Lin X, Liu Y, Ma L, Ma X, Shen L, Ma X, Chen Z, Chen H, Li D, Su Z, Chen X, Constipation induced gut microbiota dysbiosis exacerbates experimental autoimmune encephalomyelitis in C57BL/6 mice, J. Transl. Med 19 (2021) 317, 10.1186/s12967-021-02995-z. 34301274 PMC8306367

[78] Steimle A, Neumann M, Grant ET, Willieme S, De Sciscio A, Parrish A, Ollert M, Miyauchi E, Soga T, Fukuda S, Ohno H, Desai MS, Gut microbial factors predict disease severity in a mouse model of multiple sclerosis, Nat. Microbiol 9 (2024) 2244–2261, 10.1038/s41564-024-01761-3. 39009690 PMC11371644

[79] Kujawa D, Laczmanski L, Budrewicz S, Pokryszko-Dragan A, Podbielska M, Targeting gut microbiota: new therapeutic opportunities in multiple sclerosis, Gut. Microbes 15 (2023) 2274126, 10.1080/19490976.2023.2274126. 37979154 PMC10730225

[80] Zheng F, Yang Y, Lu G, Tan JS, Mageswary U, Zhan Y, Ayad ME, Lee Y-Y, Xie D, Metabolomics insights into gut microbiota and functional constipation, Metabolites. 15 (2025) 269, 10.3390/metabo15040269. 40278398 PMC12029362

[81] He W, Liu X, Wang D, Gong Y, Cui T, Zhang X, Li P, Ding X, Yang L, Zhang Q, Yang Y, Xue X, Shi L, Zhang Y, Yan Y, Post-onset intermittent fasting attenuates neuroinflammation and demyelination via a TRIB3–PERK–autophagy axis in an EAE model of multiple sclerosis, J. Neuroinflammation 22 (2025) 301, 10.1186/s12974-025-03640-y. 41299682 PMC12752437

[82] Bai M, Wang Y, Han R, Xu L, Huang M, Zhao J, Lin Y, Song S, Chen Y, Intermittent caloric restriction with a modified fasting-mimicking diet ameliorates autoimmunity and promotes recovery in a mouse model of multiple sclerosis, J. Nutr. Biochem 87 (2021) 108493, 10.1016/j.jnutbio.2020.108493. 32920091

[83] Bailey SL, Schreiner B, McMahon EJ, Miller SD, CNS myeloid DCs presenting endogenous myelin peptides “preferentially” polarize CD4+ TH-17 cells in relapsing EAE, Nat. Immunol 8 (2007) 172–180. 17206145 10.1038/ni1430

[84] Koch K, Lindner M, Fleck A-K, Liebmann M, Eschborn M, Zondler L, Díeguez-Hurtado R, Adams RH, Meyer Zu Hörste G, Zarbock A, Kuhlmann T, Wiendl H, Klotz L, CNS pericytes modulate local T cell infiltration in EAE, IJMS 23 (2022) 13081, 10.3390/ijms232113081. 36361868 PMC9658756

[85] Fletcher JM, Lalor SJ, Sweeney CM, Tubridy N, Mills KHG, T cells in multiple sclerosis and experimental autoimmune encephalomyelitis, Clin. Exp. Immunol 162 (2010) 1–11. 20682002 10.1111/j.1365-2249.2010.04143.x PMC2990924

