# Supplementary material for: Sexual dimorphism in the colonic microbiome and host’s transcriptomics profiles of a murine model of multiple sclerosis
Source: Clin Immunol Commun. Author manuscript; Available in PMC 2026 May 9. (PMC13148278; doi:10.1016/j.clicom.2026.03.003)
Supplement: MMC6 [file NIHMS2163988-supplement-MMC6.docx]

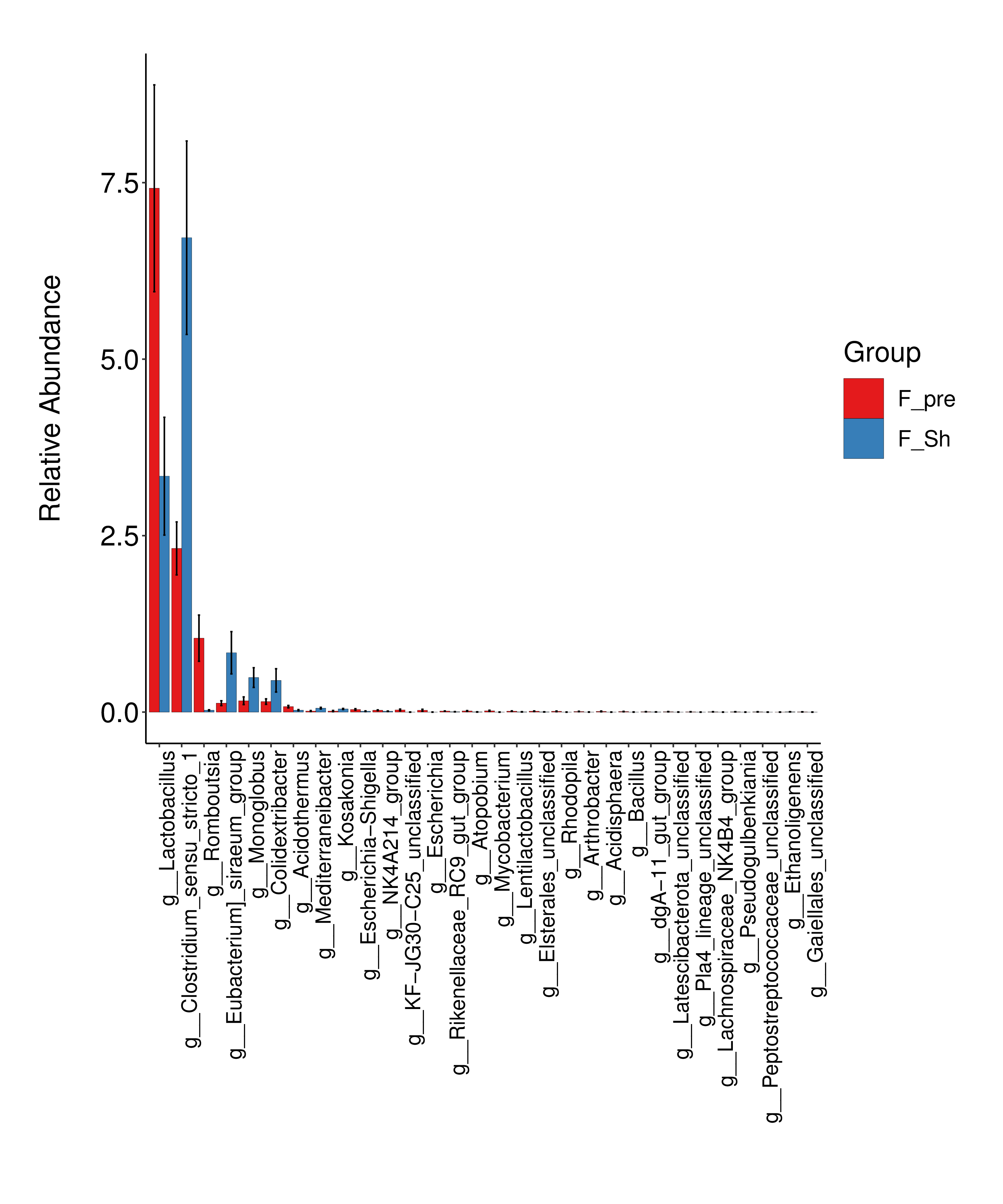


**Supplementary Figure 6.** Comparison of increased relative abundance of gut bacteria in pre-onset vs. sham females. All comparisons shown are statistically significant (*p* < 0.05) and were analyzed using the Kruskal-Wallis test. Sample sizes: Female pre-onset EAE (n = 9); Female CFA+PTX (n = 7).
