# Supplementary material for: Sexual dimorphism in the colonic microbiome and host’s transcriptomics profiles of a murine model of multiple sclerosis
Source: Clin Immunol Commun. Author manuscript; Available in PMC 2026 May 9. (PMC13148278; doi:10.1016/j.clicom.2026.03.003)
Supplement: MMC7 [file NIHMS2163988-supplement-MMC7.docx]

**
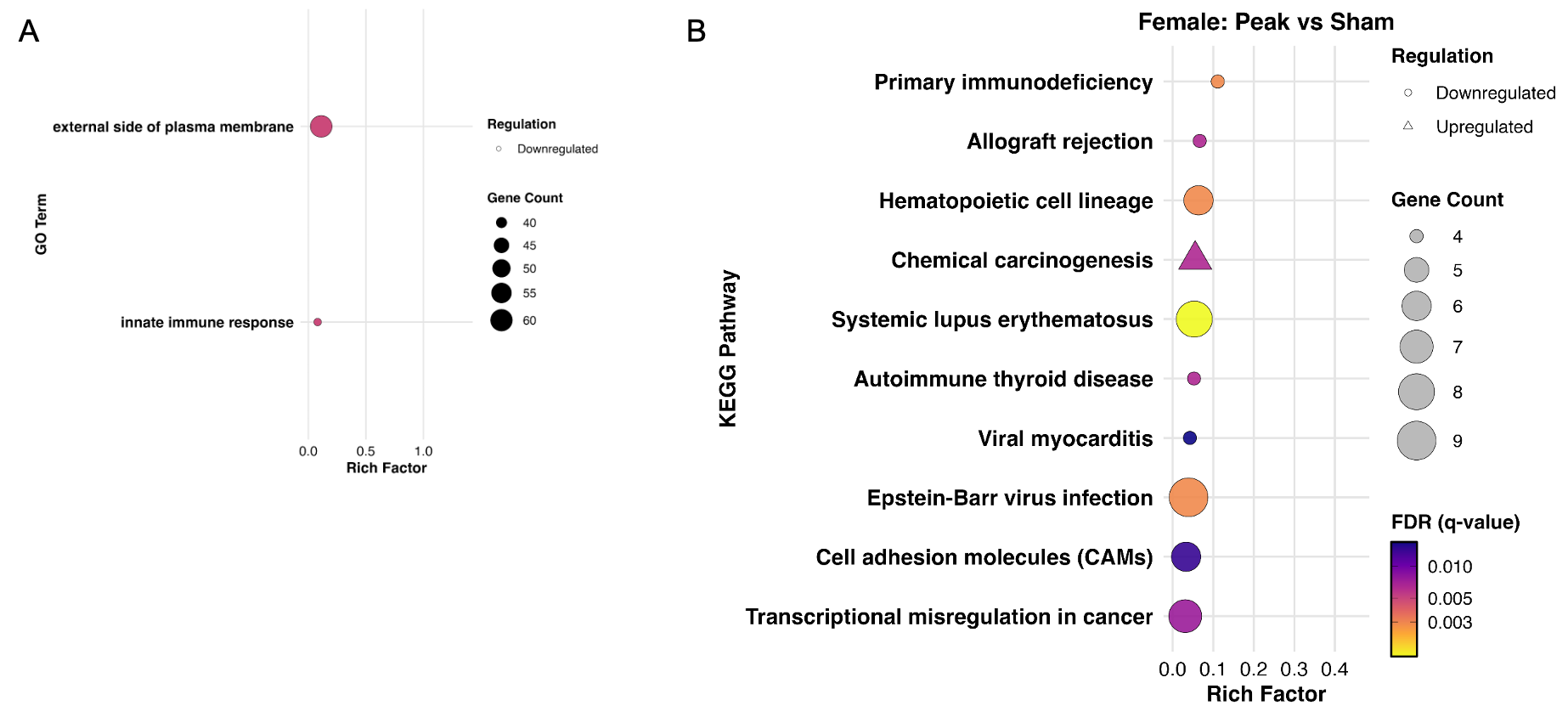
**

**Supplementary Figure 7.** Female colon pre-onset vs. sham. A) GO enrichment scatterplots. B) KEGG pathway enrichment scatterplots. Sample sizes: Female pre-onset EAE (n = 9); Female CFA+PTX (n = 7).
