# Supplementary material for: Sexual dimorphism in the colonic microbiome and host’s transcriptomics profiles of a murine model of multiple sclerosis
Source: Clin Immunol Commun. Author manuscript; Available in PMC 2026 May 9. (PMC13148278; doi:10.1016/j.clicom.2026.03.003)
Supplement: MMC5 [file NIHMS2163988-supplement-MMC5.docx]

**Supplementary Figure 5
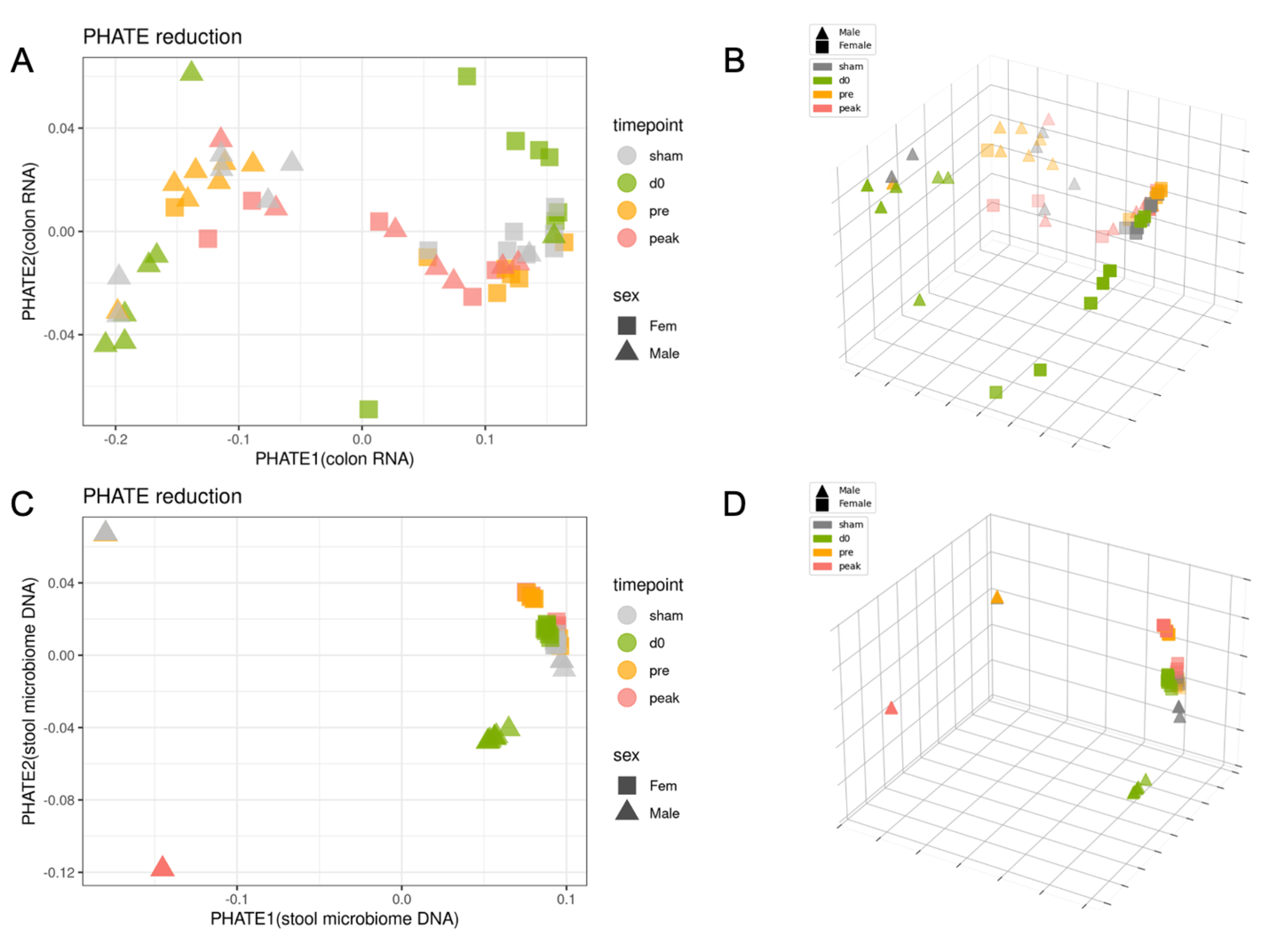
**

**Supplementary Figure 5.** PHATE reduction and visualization of RNA-seq and 16S rRNA microbiome sequencing independently. Colon RNA sequencing using PHATE reduction in 2D (A) and 3D (B). Microbiome 16s rRNA sequencing using PHATE reduction in 2D (C) and 3D (D).
