# Supplementary material for: Sexual dimorphism in the colonic microbiome and host’s transcriptomics profiles of a murine model of multiple sclerosis
Source: Clin Immunol Commun. Author manuscript; Available in PMC 2026 May 9. (PMC13148278; doi:10.1016/j.clicom.2026.03.003)
Supplement: MMC3 [file NIHMS2163988-supplement-MMC3.docx]

**Supplementary Figure 3**

**
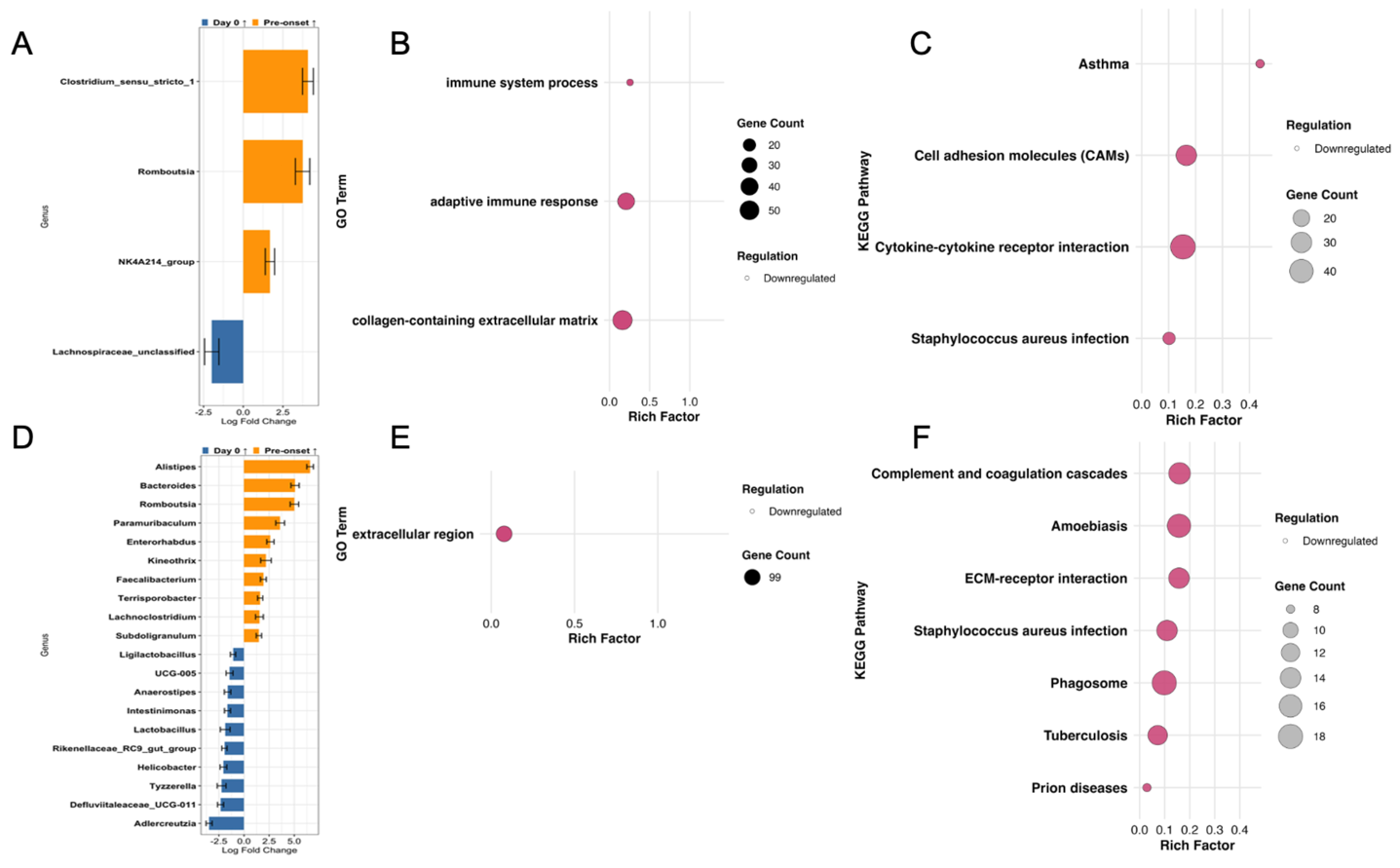
**

**Supplementary Figure 3.** Colonic microbiome differential abundance (genus) and colon RNA-sequencing, comparing D0 to pre-onset EAE in males and females. (A) Female colonic microbiome abundance of D0 and pre-onset EAE. Positive log₂ fold changes indicate genera enriched in the female D0 EAE group, and negative values indicate enrichment in the female pre-onset EAE group. Only genera with FDR < 0.05 are shown; colon RNA-sequencing analysis using (B) GO and (C) KEGG. Only significant enrichment, FDR < 0.05, was displayed. The shape of the point indicates regulation (triangle: upregulation; circle: downregulation), the point size corresponds to gene count, and the point coloration corresponds to the FDR value. (D) Male colonic microbiome abundance comparing D0 to pre-onset EAE. Positive log₂ fold changes indicate genera enriched in the male D0 EAE group, and negative values indicate enrichment in the male pre-onset EAE group. Only genera with a false discovery rate FDR < 0.05 are shown. Colon RNA-sequencing analysis using (E) GO and (F) KEGG. Only significant enrichment, FDR < 0.05, was displayed. The shape of the point indicates regulation (triangle: upregulation; circle: downregulation), the point size corresponds to gene count, and the point coloration corresponds to the FDR value.
