# Supplementary material for: Sexual dimorphism in the colonic microbiome and host’s transcriptomics profiles of a murine model of multiple sclerosis
Source: Clin Immunol Commun. Author manuscript; Available in PMC 2026 May 9. (PMC13148278; doi:10.1016/j.clicom.2026.03.003)
Supplement: MMC1 [file NIHMS2163988-supplement-MMC1.docx]

**Supplementary Figure 1**


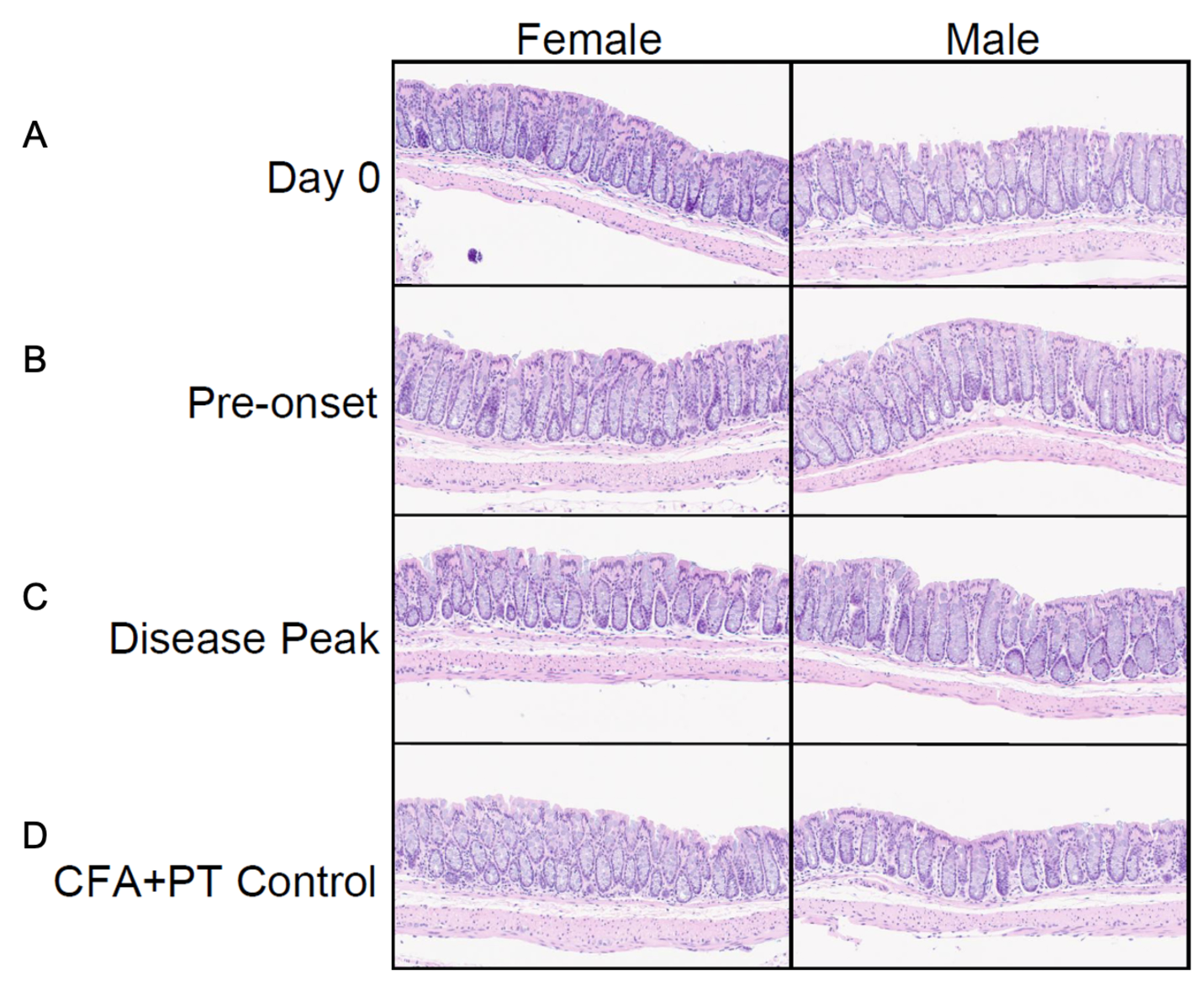


**Supplementary Figure 1.** H&E staining of the colon of male and female mice throughout EAE progression (20x magnification); Male and female on day 0 (A), pre-onset (B), disease peak (day 17/19 for female/male) (C), and CFA+PT control (D).
