# Supplementary material for: Sexual dimorphism in the colonic microbiome and host’s transcriptomics profiles of a murine model of multiple sclerosis
Source: Clin Immunol Commun. Author manuscript; Available in PMC 2026 May 9. (PMC13148278; doi:10.1016/j.clicom.2026.03.003)
Supplement: MMC2 [file NIHMS2163988-supplement-MMC2.docx]

**Supplementary Figure 2**


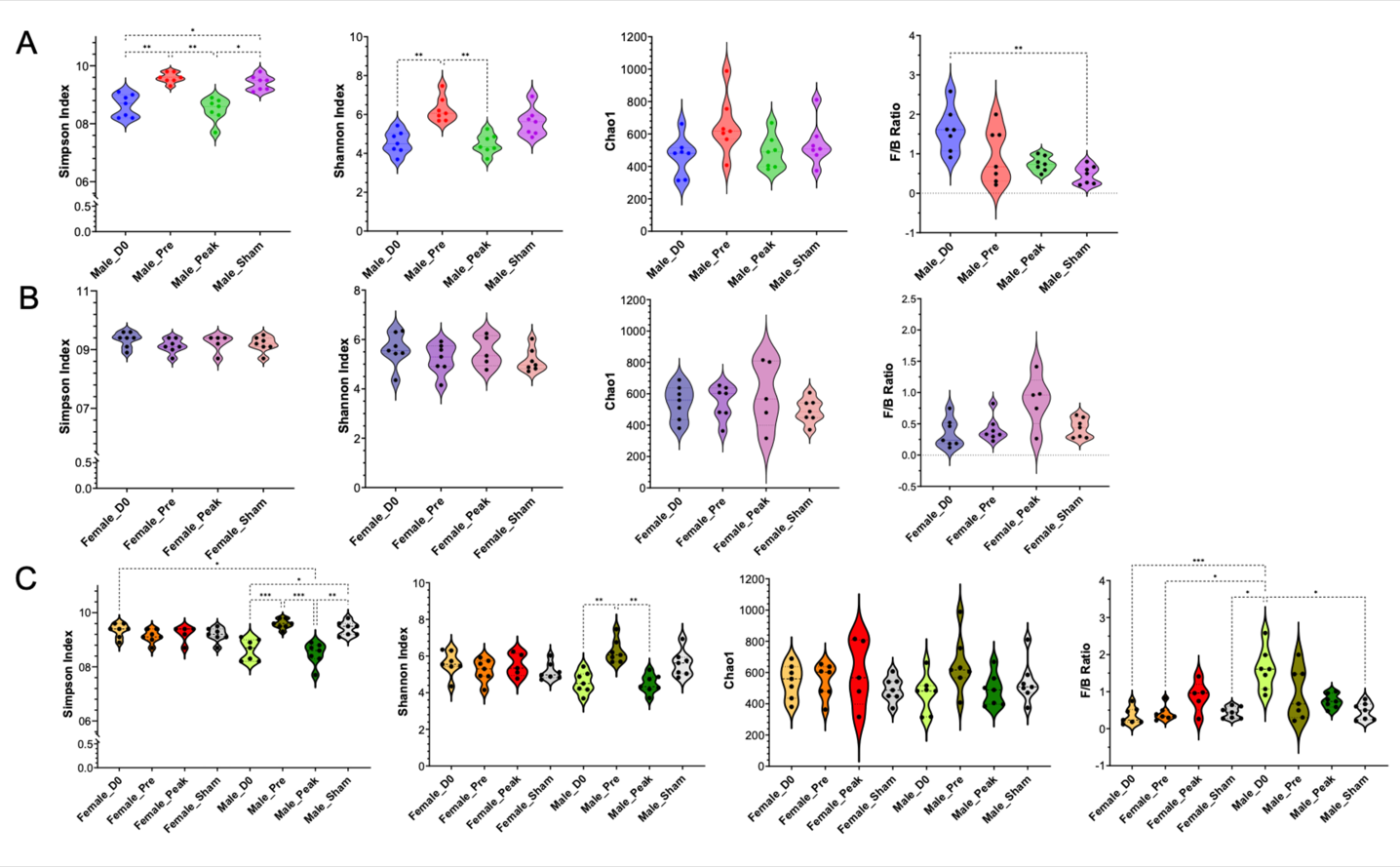


**Supplementary Figure 2.** Colonic microbiome diversity across all timepoints was analyzed separately for males and females, and combined. (A) Male colonic microbiome comparison with Simpson, Shannon, Chao1, and F/B ratio. (B) Female colonic microbiome comparison with Simpson, Shannon, Chao1, and F/B ratio. (C) Comparison of male and female (separate and combined) colonic microbiomes using Simpson, Shannon, Chao1, and F/B ratio. Two-way ANOVA followed by Kruskal-Wallis: *, *p* < 0.05; **, *p* < 0.01; ***, *p* < 0.001.
